# Strategies for Primary Prevention of Coronary Heart Disease Based on Risk Stratification by the ACC/AHA Lipid Guidelines, ATP III Guidelines, Coronary Calcium Scoring, and C-Reactive Protein, and a Global Treat-All Strategy: A Comparative--Effectiveness Modeling Study

**DOI:** 10.1371/journal.pone.0138092

**Published:** 2015-09-30

**Authors:** Benjamin Z. Galper, Y. Claire Wang, Andrew J. Einstein

**Affiliations:** 1 Brigham and Women’s Hospital, Division of Cardiology, Boston, Massachusetts, United States of America; 2 Columbia University Medical Center, Mailman School of Public Health, New York, New York, United States of America; 3 Columbia University Medical Center and New York-Presbyterian Hospital, Department of Medicine, New York, New York, United States of America; 4 Columbia University Medical Center and New York-Presbyterian Hospital, Division of Cardiology, New York, New York, United States of America; 5 Columbia University Medical Center and New York-Presbyterian Hospital, Department of Radiology, New York, New York, United States of America; University of Milan, ITALY

## Abstract

**Background:**

Several approaches have been proposed for risk-stratification and primary prevention of coronary heart disease (CHD), but their comparative and cost-effectiveness is unknown.

**Methods:**

We constructed a state-transition microsimulation model to compare multiple approaches to the primary prevention of CHD in a simulated cohort of men aged 45–75 and women 55–75. Risk-stratification strategies included the 2013 American College of Cardiology/American Heart Association (ACC/AHA) guidelines on the treatment of blood cholesterol, the Adult Treatment Panel (ATP) III guidelines, and approaches based on coronary artery calcium (CAC) scoring and C-reactive protein (CRP). Additionally we assessed a treat-all strategy in which all individuals were prescribed either moderate-dose or high-dose statins and all males received low-dose aspirin. Outcome measures included CHD events, costs, medication-related side effects, radiation-attributable cancers, and quality-adjusted-life-years (QALYs) over a 30-year timeframe.

**Results:**

Treat-all with high-dose statins dominated all other strategies for both men and women, gaining 15.7 million QALYs, preventing 7.3 million myocardial infarctions, and saving over $238 billion, compared to the status quo, far outweighing its associated adverse events including bleeding, hepatitis, myopathy, and new-onset diabetes. ACC/AHA guidelines were more cost-effective than ATP III guidelines for both men and women despite placing 8.7 million more people on statins. For women at low CHD risk, treat-all with high-dose statins was more likely to cause a statin-related adverse event than to prevent a CHD event.

**Conclusions:**

Despite leading to a greater proportion of the population placed on statin therapy, the ACC/AHA guidelines are more cost-effective than ATP III. Even so, at generic prices, treating all men and women with statins and all men with low-dose aspirin appears to be more cost-effective than all risk-stratification approaches for the primary prevention of CHD. Especially for low-CHD risk women, decisions on the appropriate primary prevention strategy should be based on shared decision making between patients and healthcare providers.

## Introduction

Cardiovascular disease, and in particular coronary heart disease (CHD), is now the leading cause of death and disability-associated life years both globally and in the United States [1,2]. While previous guidelines, such as the National Cholesterol Education Program’s Adult Treatment Panel (ATP) III and the European Association of Cardiology and the European Atherosclerosis Society guidelines, focused on treating individuals to a target LDL-C, other approaches, including the recently released 2013 American College of Cardiology/American Heart Association (ACC/AHA) Guideline on the Treatment of Blood Cholesterol to Reduce Atherosclerotic Cardiovascular Risk in Adults, have recommended treating a person with statins based on their global CHD or atherosclerotic cardiovascular disease (ASCVD) risk [3–8]. With the emergence of new techniques to assess risk-stratification for CHD such as coronary artery calcium (CAC) scanning and inflammatory biomarkers such as C-reactive protein (CRP), there may be promise for more cost-effective strategies to identify asymptomatic patients who would benefit from treatments to prevent coronary heart disease (CHD) [9,10].

While Framingham Risk Scores (FRS) have been demonstrated to correlate well with future risk of CHD [11], recent studies have demonstrated that CAC and CRP can help further classify intermediate-risk patients [9,10,12,13]. However, none of these risk-stratification tools, including FRS, have been studied to determine whether they directly decrease the incidence of new CHD events. Nevertheless, multiple approaches have been proposed to address the risk stratification of individuals for CHD. The Screening for Heart Attack Prevention and Education (SHAPE) guidelines recommend nationwide CAC screening that would include most of the adult population of the United States [14]. In 2009, the State of Texas mandated payers to provide coverage for CAC screening of patients with FRS>10% [15,16]. Moreover, the Justification for the Use of Statins in Primary Prevention (JUPITER) trial demonstrated that the use of rosuvastatin in patients with normal low-density lipoprotein cholesterol (LDL-C) but CRP≥2.0 mg/L can decrease risk of new CHD events by 54%, compared to the FRS-based treatment guidelines of the ATP III program [10,17]. Recently-released ACC/AHA guidelines on the treatment of cholesterol move away from treating a person based on reaching a goal LDL-C and utilize the Pooled Cohort Risk Equation (PCE) instead of FRS to determine a person’s global ASCVD risk. These guidelines initiate statin treatment if a person is diabetic or has an LDL-C > 190 mg/dl, but, unlike ATP III, treatment for non-diabetics with an LDL-C < 190 is initiated based solely on the risk of future cardiovascular disease and does not utilize a threshold LDL-C level to determine the need to initiate treatment or a target LDL-C to achieve once treatment has been started [7–8]. On the other hand, with the advent of generic-priced statins, there is active debate regarding the utility of risk-stratification and some have suggested that treating all intermediate risk persons with high-intensity statins, regardless of LDL-C or FRS level, is the most cost-effective approach while others have been concerned that placing an entire population on high-intensity statins would expose them to an increased risk of statin-related adverse events [18–21]. While initial analyses have demonstrated that risk-stratification using either CAC or CRP could be cost-effective [22–24], no comprehensive cost-effectiveness analysis has been performed to systematically compare all these approaches in an era of generic-priced statins. Additionally, no analysis has evaluated the global use of high-dose statins to prevent CHD in the primary prevention population. In this study, we used a decision-analytic model to compare the cost-effectiveness of these multiple approaches aimed at reducing the burden of CHD among the asymptomatic US adult population.

## Methods

### Model

**Fig 1:**
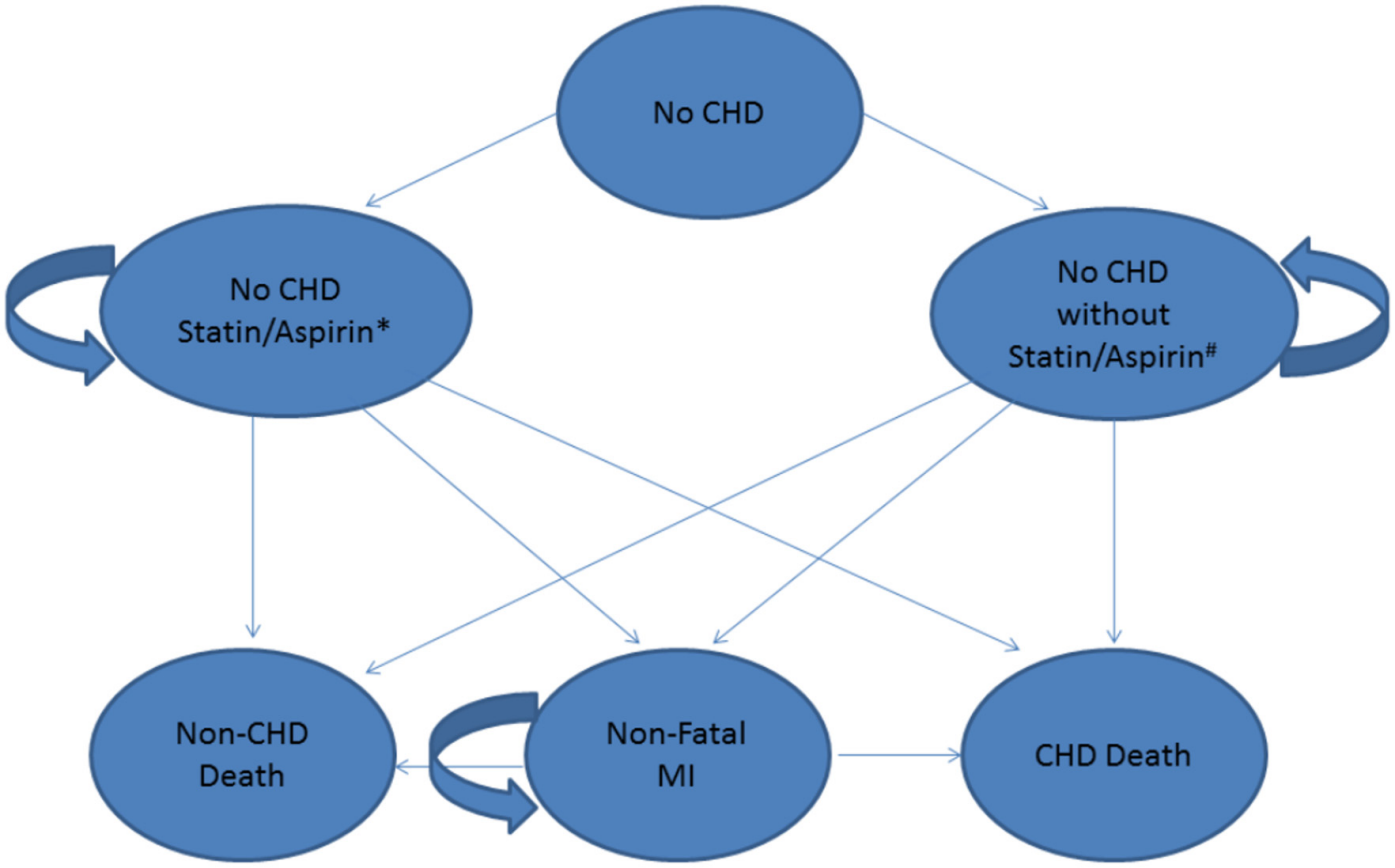
State-transition diagram for Markov model. CHD = coronary heart disease. MI = myocardial infarction.

We constructed a state-transition (Markov) microsimulation model in TreeAge Pro Suite 2009 (TreeAge Software, Inc., Williamstown, MA). The model evaluated the target population, suggested in SHAPE, of US men aged 45–75 and women aged 55–75 who are asymptomatic defined as having no prior CHD event, which would include myocardial infarction or coronary revascularization as well not having a CHD equivalent such as diabetes. It was treated as a closed-cohort population and individuals were followed in the model until either their death or for 30 years. In 2010, this population would represent 46.9 million men and 27.3 million women [25]. Baseline values of FRS, LDL-C, CAC, and CRP were generated for each simulated individual based on published distributions [3,26,27]. Depending on age, sex, and FRS, each year an individual faces a probability of developing a CHD event and transitions to the post-CHD states or dying from other causes. Under different screening strategies, individuals designated as candidates for treatment receive medications (moderate- or high-dose statins and low-dose aspirin), incur relevant treatment costs, and alter subsequent CHD risks. Average costs and quality-of-life adjustments were incorporated for each health state and event. One million men and women were simulated and results were then extrapolated for the total population. Over the period 2011–2040, the model estimated the total number of events under each strategy. Outcomes compared were the incidence of new CHD, which included both fatal MI and non-fatal MI, all-cause mortality, costs, quality adjusted life years (QALY) gained, side-effects, resultant additional costs from medications prescribed, and incidence of radiation-attributable malignancies from CAC (using a typical protocol with an effective dose of 2.3 mSv) [28]. Fig 1 demonstrates the possible transition states that an individual can be placed in depending on their risk factors which are modified in our model by the use of statin and aspirin therapy. In order to characterize the joint uncertainties from key parameters, we estimated the 95% confidence intervals using probabilistic sensitivity analyses (PSA), in which we simultaneously varied the assumed CHD risk reduction from aspirin and statin therapies, disutilities from aspirin and statin therapies, radiation doses from CAC, and the costs of CAC, aspirin, and statins.

### Strategies

**Fig 2:**
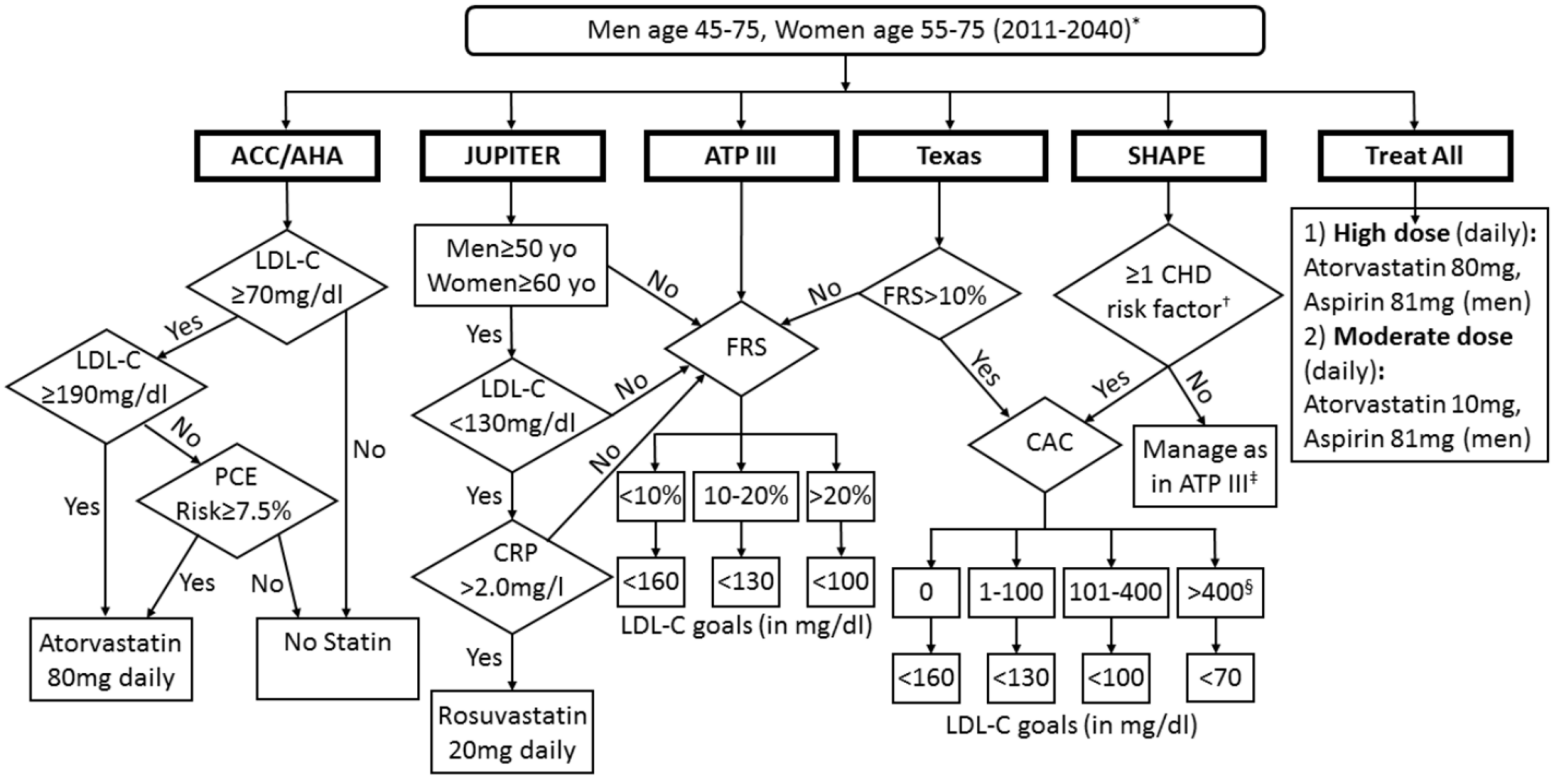
Flow-diagram of all strategies simulated. *All persons with Framingham Risk Score >10% received aspirin 81mg daily, except for treat-all in which all men received aspirin 81mg. †SHAPE treats the following as risk factors: total cholesterol >200mg/dl, blood pressure >120/80, diabetes mellitus, smoking, family history of CHD, and metabolic syndrome. ‡SHAPE considers individuals without any of its specified risk factors to be “very low risk” and treats this as an “exit” from its screening algorithm, without calcium scoring being performed. We assumed such individuals not undergoing calcium scoring would then be treated to LDL-C goals in accordance with the approach of ATP III; in general such individuals have Framingham risk of <10%. §Per SHAPE, all persons with CAC >400 underwent nuclear stress testing followed by diagnostic coronary angiography and revascularization if indicated based on stress testing results in simulations. If nuclear stress testing is negative persons are treated to goal LDL-C of <70mg/dl. JUPITER = Justification for the Use of Statins in Primary Prevention. ATP III = Adult Treatment Panel III. SHAPE = Screening for Heart Attack Prevention and Education. YO = Years-Old. LDL-C = Low-density lipoprotein. CRP = C-Reactive Protein. FRS = Framingham Risk Score. CAC = Coronary Artery Calcium Score. PCE = pooled cohort risk equation. Other abbreviations same as in prior figure.

Eight strategies were compared (Fig 2 and Table 1): (1) the status quo use of statin and aspirin therapy in the primary prevention population, which served as the comparator to all other strategies [29]; (2) an ATP III approach which assigned statin therapy based on FRS, and LDL-C level [30]; (3) an ACC/AHA guidelines approach in which all people with an LDL-C > 190 mg/dl were placed on high-dose statins and those with an LDL-C between 70 and 190 mg/dl and a PCE 10-year risk of ≥7.5% were placed on high-intensity statins [7–8]; (4) a SHAPE approach in which CAC scores determine therapy for all people who had one or more CHD risk factors [14]; (5) the approach of the Texas Heart Attack Prevention Law (referred to subsequently as Texas), in which only patients with FRS>10% undergo CAC to determine therapy and those with FRS<10% were treated based on ATP III [15,16]; (6) a JUPITER approach in which all men 50 years or older and women 60 years or older with LDL-C<130 mg/dl and CRP>2 mg/L receive 20mg of Rosuvastatin [10]; (7) treating everyone with moderate-dose statins and all men with aspirin 81mg daily; (8) and treating everyone with high-dose statins and all men with aspirin 81mg daily. Except for the JUPITER strategy, we assumed that all patients assigned to high-dose statins received 80mg of atorvastatin. All risk stratification (i.e., non-treat-all) strategies, excluding status quo, included low-dose aspirin for men with FRS>10%. For the CAC- and CRP-based strategies any person who did not meet inclusion criteria for each strategy was treated based on the ATP III guidelines.

**Table 1:** Inclusion and Exclusion Criteria as Well as Overview of each Approach to Primary Prevention Strategies Evaluated in the Model.

**ATP III**
** Inclusion Criteria—treat with appropriate dose of Atorvastatin to reach LDL goal:**
- Men aged 45–75 yo, or Women aged 55–75 yo
-If FRS < 10%, LDL-C goal of < 160mg/dl
-If FRS 10–20%, LDL-C goal of < 130mg/dl
-If FRS >20%, LDL-C goal of < 100mg/dl
**No Patients Excluded**
Aspirin 81mg for all men with an FRS of > 10%
**ACC/AHA**
**Inclusion Criteria—treat with 80mg of Atorvastatin daily if:**
- Men aged 45–75 yo, or Women aged 55–75 yo
-LDL-C 70-190mg/dl and PCE risk of ≥ 7.5%
**Exclusion Criteria**
-LDL < 70 mg/dl (no statin)
-LDL > 190mg/dl (Atorvastatin 80mg daily regardless of PCE risk)
Aspirin 81mg for all men with an FRS of > 10%
**JUPITER**
**Inclusion Criteria—treat with 20mg of Rosuvastatin daily if:**
-Men aged 50–75 yo, or Women aged 60–75 yo
-LDL-C < 130mg/dl
-CRP > 2.0mg/L
**Exclusion Criteria—treat based on ATP III guidelines (see above)**
-If inclusion criteria not met treat based on LDL goals for a given FRS
Aspirin 81mg for all men with an FRS of > 10%
**SHAPE**
**Inclusion Criteria—treat with Atorvastatin to reach LDL goal for given CAC score:**
-Men aged 50–75 yo, or Women aged 60–75 yo
-If at least 1 CHD risk factor* will undergo CAC testing and if:
-CAC = 0, LDL-C goal of < 160mg/dl
-CAC 1–100, LDL-C goal of < 130mg/dl
-CAC 100–400, LDL-C goal of < 100mg/dl
-CAC > 400, LDL-C goal < 70mg/dl and undergo nuclear stress test#
**Exclusion Criteria—treat based on ATP III guidelines (see above)**
-If men < 50 yo or women < 60 yo or
-If no CHD risk factors
Aspirin 81mg for all men with an FRS of > 10%
**Texas**
**Inclusion Criteria—treat based on SHAPE guidelines (see above)**
-Men aged 50–75 yo, or Women aged 60–75 yo
-If FRS > 10% undergo CAC test and treat based on LDL-C goal for given CAC
**Exclusion Criteria—treat based on ATP III guidelines (see above)**
-If men < 50 yo or women < 60 yo or
-If FRS < 10%
Aspirin 81mg for all men with an FRS of > 10%
**Treat-All High-Dose Statins**
**Inclusion Criteria-treat with Atorvastatin 80mg daily**
-All Men aged 45–75 yo, Women aged 55–75 yo regardless of risk
**No patients excluded**
Aspirin 81mg for all men with an FRS of > 10%
**Treat-All Moderate-Dose Statins**
**Inclusion Criteria-treat with Atorvastatin 10mg daily**
-All Men aged 45–75 yo, Women aged 55–75 yo regardless of risk
**No patients excluded**
Aspirin 81mg for all men with an FRS of > 10%

* SHAPE treats the following as risk factors: total cholesterol >200mg/dl, blood pressure >120/80, diabetes mellitus, smoking, family history of CHD, and metabolic syndrome.

^#^ Stress test deemed positive if >10% of myocardium demonstrates reversible ischemia. Positive stress test would lead to coronary angiography and if indicated coronary revascularization.

JUPITER = Justification for the Use of Statins in Primary Prevention

ATP III = Adult Treatment Panel III

SHAPE = Screening for Heart Attack Prevention and Education

YO = Years-Old

LDL-C = Low-density lipoprotein

CRP = C-Reactive Protein

FRS = Framingham Risk Score

CAC = Coronary Artery Calcium

PCE = pooled cohort risk equation

For the 2013 ACC/AHA guidelines strategy, in order to determine the PCE distribution of 10-year ASCVD risk in our patient population we utilized the known correlation of FRS to Pooled Cohort risk as demonstrated in the appendix to the 2013 guidelines (Table A in S1 File) [8,30–32]. In order to establish a person’s Pooled Cohort 10-year risk in our model, we converted a person’s FRS to a Pooled Cohort risk score. Additionally, while all other strategies investigated in our model excluded patients with diabetes, the ACC/AHA guidelines include diabetics. We did not include diabetics in the ACC/AHA strategy since substantial evidence demonstrates and current practice recommends that all diabetics regardless of risk be treated with statin therapy, and thus regardless of a diabetic’s Pooled Cohort risk they would be recommended to take statins [33–34]. Lastly, while the ACC/AHA guidelines recommend initiating risk stratification for both men and women at age 40, the ACC/AHA guidelines themselves question the utility of starting risk stratification so early in one’s life as there is little data on the correlation of 10-year CHD risk in a 40 year old and cardiovascular event risk beyond 10 years [7]. Indeed, a recent analysis demonstrated that the ACC/AHA guidelines would have a minimal effect in reclassifying the 40 to 59 year old population as being at higher risk as compared to ATP III, but when applying the ACC/AHA guidelines to 60 to 75 year olds, 77.3% of the population would now be recommended to take statin therapy as compared to 47.8% if the ATP III strategy were used [35]. We therefore utilized the same age threshold of 45 years-old in men and 55 years-old in women for the ACC/AHA guideline strategy as we used for other strategies evaluated. Lastly, we did not include repeat testing for patients in any of the risk-stratification approaches.

### Data Inputs and Assumptions

Table 2 summarizes key input parameters associated with the simulated population [10,14,17,19,22,24,28,29,36–70]. Baseline age distribution was based on 2009 US census data [37]. Annual mortality rates, by age and sex, were obtained from US vital statistics (Table B in S1 File). Assignment of FRS was based on published population-representative FRS distributions (Table C in S1 File), and baseline LDL-C levels were assigned conditional on sex and FRS (Table D in S1 File) [3,18,29]. For SHAPE and Texas, the distribution of CAC scores was based on each individual’s age and gender [26]. Since statistically significant reduction in the primary prevention of CHD events with aspirin therapy has only been demonstrated in men and not in women, we only prescribed aspirin for men in our simulations [40]. Additionally, since the benefit of aspirin therapy for primary prevention is more clearly defined in persons with more FRS-defined risk factors, we assigned aspirin therapy only to men with FRS>10%, except for treat-all simulations where all men received aspirin [71]. All costs were assumed based on 2010 baseline costs in US dollars. Costs for screening and diagnostic tests such as CAC scanning and CRP tests were obtained from Medicare reimbursement rates or published literature [15,39,47]. Costs of statin therapy for all interventions evaluated were the sum of the wholesale price of an annual supply of generic atorvastatin, and the cost of additional annual physician visits and laboratory blood work, including liver function testing, beyond the usual outpatient healthcare utilization that would be expected for patients undergoing general primary care who were not taking statin therapy [18,39,47]. The cost of a one-year supply of Rosuvastatin was assumed to be high (mean $1,325) until 2016, after which its patent expires and we assumed the same price as generic atorvastatin [41].

**Table 2:** Selected Inputs to the State-Transition Model.

Input variable	Primary simulation assumption (range for Probabilistic Sensitivity Analysis)*	Reference
**Aspirin**		
Dose	81mg	
RR of CHD§, men	0.77 (0.67–0.89)*	40
RR of CHD§, women	1	40
RR of ischemic stroke, men and women	1	40
Cost	$7.00 ($2-$16)^†^	41
Adverse Events		
Gastrointestinal bleeding		
Annual incidence	0.000142 (0-.00036)*	42
QALY decrement per episode	0.06	42
Cost per episode	$4,100	42
Hemorrhagic Stroke		
Annual incidence	0.0002	42
QALY decrement per episode	0.351	42
Cost per episode	$35,618	39
Percent aspirin use “status quo”^#^ men		19
Age 35–44	16.1%	
Age 45–54	28.8%	
Age 55–64	44.1%	
Age 65–74	53.7%	
Age 75–99	55.1%	
Percent aspirin use “status quo”^#^ women		19
Age 35–44	11.1%	
Age 45–54	22.6%	
Age 55–64	37.9%	
Age 65–74	47.5%	
Age 75–99	50.8%	
**Statin**		
Percent statin use “status quo”^#^ men		29
Age 35–44	4.8%	
Age 45–54	11.4%	
Age 55–64	30.1%	
Age 65–74	37.7%	
Age 75–84	30.3%	
Percent statin use “status quo”^#^ women		29
Age 35–44	3.0%	
Age 45–54	7.2%	
Age 55–64	18.6%	
Age 65–74	32.9%	
Age 75–84	29.9%	
Reduction in CHD risk per each 1mg/dl in LDL-C	0.6%	34
Moderate-dose therapy		
Medication	Atorvastatin 10mg	
Decrease in LDL-C (mg/dl)	34% (27%-38%)*	18,49–57
Annual Cost^^^	$83.81 ($35.03-$189.55)^†^	18,39
Overall Disutility from statin use	0.00023 (0.00010-.00049)*	18,49–57
Statin-associated adverse events		
Hepatitis		
Without Liver Failure		
Annual incidence	0.00675 (.0054-.0081)*	49–53
QALY decrement per episode	0.000035	18
Cost per episode	$36.39 ($6.00-$90.00) ^†^	18,49–53
With Liver Failure		
Annual incidence	0.0000305 (.000019-.0000325)	18,49–53
QALY decrement per episode	0.0262	18
Cost per episode	$15,729 ($9,800-$21,600) ^†^	18,39
Myopathy		
Without rhabdomyolysis		
Annual incidence	0.0037 (.0031-.0043)*	49–53
QALY decrement per episode	0.0164	18
Cost per episode	$28.41 ($4.70-$71.00) ^†^	18,39
With rhabdomyolysis		
Annual incidence	0.0000295 (0–0.000145)*	49–53
QALY decrement per episode	0.0852	18
Cost per episode	$11,745 ($6,800-$16,600) ^†^	18,49–53
New Statin Related Diabetes		
Annual incidence	0.001 (0.0004–0.0022)*	54,55
QALY decrement per episode	0.12 (0.1–0.14)*	56
Cost per episode	$6,357 ($3050-$9,500) ^†^	22
Statin Intolerance		
Annual incidence	0.175	57,70
QALY decrement per episode	0.00028 (0.000068–0.00055)*	24
High-dose therapy		
Medication	Atorvastatin 80mg	
Decrease in LDL-C (mg/dl)	55% (51%-59%)*	18,49–57
Annual Cost^^^	$85.38 ($35.28-$195.77)^†^	18
Overall Disutility from statin use	0.00054 (0.00024–0.0012)*	18,49–57
Statin-associated adverse events		
Hepatitis		
Without Liver Failure		
Annual incidence	0.0135 (0.011–0.016)*	49–53
QALY decrement per episode	0.000035	18
Cost per episode	$36.39 ($6.00-$90.00) ^†^	18,49–53
With Liver Failure		
Annual incidence	0.000061 (0.000058–0.000065)*	18,49–53
QALY decrement per episode	0.0262	18
Cost per episode	$15,729 ($9,800-$21,600) ^†^	18,39
Myopathy		
Without rhabdomyolysis		
Annual incidence	0.0074 (0.0062–0.0086)*	49–53
QALY decrement per episode	0.0164	18
Cost per episode	$28.41 ($4.70-$71.00) ^†^	18,39
With rhabdomyolysis		
Annual incidence	0.000059 (0–0.00029)*	49–53
QALY decrement per episode	0.0852	18
Cost per episode	$11,745 ($6,800-$16,600) ^†^	18,49–53
New Statin Related Diabetes		
Annual incidence	0.003 (0.0012–0.0067)*	54,55
QALY decrement per episode	0.12 (0.1–0.14)*	56
Cost per episode	$6,357 ($3050-$9,500) ^†^	22
Statin Intolerance		
Annual incidence	0.175	57,70
QALY decrement per episode	0.00028 (0.000068–0.00055)*	24
**Annual costs for patients on statins**		
Physician visits	$65.30	39
Laboratory testing	$30.32	47
**Discount rate**	0.03	69
**Myocardial Infarction**		
Cost, upfront	$25,567	29,36
Cost, annual	$3,109	29,36
Utility, first 8 days	0.829	58
Annual Utility	0.865	58
**Calcium Scoring**		
RR (95% CI) of CHD by Calcium Score		43
Calcium Score 0	1	
Calcium Score 1–100	1.9 (1.3–2.8)	
Calcium Score 101–400	4.3 (3.1–6.1)	
Calcium Score 401–1000	7.2 (5.2–9.9)	
Calcium Score > 1000	10.8 (4.8–27.7)	
Radiation doses from one CAC scan		28
Effective dose	2.3 mSv	
Lung equivalent dose	6.4 mSv	
Breast equivalent dose, females	7.7 mSv	
Bone marrow equivalent dose	1.2 mSv	
Cancer Risks per 100,000, 1 scan		28
Overall Cancer Risk	1 (0.09–2.17)^†^	
Lung Cancer, 45 year old male	6.528	
Lung Cancer, 55 year old female	15.04	
Breast Cancer, 55 year old female	3.88	
Leukemia, 45 year old male	1.01	
Leukemia, 55 year old female	0.72	
Minimum lag-time from CAC scan to cancer		28
Solid tumors	10 years	
Leukemia	2 years	
Lifetime cost of Cancer		39
Lung	$56,624	
Breast	$37,306	
Leukemia	$98,000	
QALY for Cancer		
Lung, 1^st^ year after diagnosis	0.42	45
Lung, after 1^st^ year	0.65	45
Breast	0.7	45
Leukemia, first 3 years	0.85	46
Leukemia more than 3 years	0.56	46
Cost of CAC scan	$200 ($100-$400) ^†^	15
Nuclear Stress Test		
Cost	$878.42	39
QALY decrement from test	0.0006	45,59,60
Increased incidence of cancer per test	0.000045	60,61
Cost per cancer	$93,777	62
QALY lost per cancer	12.3	45,46
% with minimal ischemia (< 4.9%)	43%	63
% with mild ischemia (5–9.9%)	26%	63
% with moderate/severe ischemia (> 10%)	31%	64
% moderate/severe ischemia w PCI	33%	64
% moderate/severe ischemia w CABG	19%	65
Annual mortality moderate/severe ischemia	5.2%	65
Annual rate of MI moderate/severe ischemia	3.1%	66
Reduction in mortality w revascularization	2.3%	66
Reduction in MI w revascularization	1.4%	66
Cost of diagnostic angiography	$2,585	39
Cost of PCI including complications	$16,795	39
Cost of CABG	$44,820	39
QALY decrement from PCI	0.14 first month, 0 after	67,68
QALY decrement from CABG	0.16 first 2.5 month, 0 after	67,68
**JUPITER**		
Median CRP level by FRS (95% CI)		
Men		27
FRS 0–5%	0.9 mg/L (0.5–2)	
FRS 6–10%	1.1 mg/L (0.5–2)	
FRS 11–15%	1.7 mg/L (1–3.4)	
FRS 16–23%	2.0 mg/L (1–4)	
FRS >23%	2.5 mg/L (1.2–4.6)	
Women		27
FRS 0–5%	1.9 mg/L (0.6–4.6)	
FRS 6–10%	1.9 mg/L (0.6–4.2)	
FRS 11–15%	2.5 mg/L (1.5–4.8)	
FRS 16–20%	3.1 mg/L (1.8–5.6)	
FRS >20%	3.8mg/L (1.7–7.6	
Cost of CRP Test	$19	47
Rosuvastatin 20mg		
Cost, through 2016	$1,341 ($898-$1,735)^†^	41
Cost, after 2016	$83 ($34.90-$186.40)^†^	18
LDL-C Reduction	47mg/dl	10
CHD risk reduction per 1mg/dl in LDL-C	0.6%	34
RR of CHD based on CRP and FRS		17
FRS < 10%		
CRP< 0.5 mg/L	1	
CRP 0.5–1 mg/L	1.9	
CRP 1–3 mg/L	2.0	
CRP 3–10 mg/L	3.1	
CRP >10 mg/L	4.5	
FRS > 10%		17
CRP< 0.5 mg/L	1	
CRP 0.5–1 mg/L	1.2	
CRP 1–3 mg/L	2.2	
CRP 3–10 mg/L	2.5	
CRP >10 mg/L	4.8	

*beta distribution for probabilistic sensitivity analysis

^†^log-normal distribution for probabilistic sensitivity analysis

^#^The status quo simulation represents outcomes based on current statin and aspirin use in the US primary prevention population

^^^Includes costs of adverse events

CABG = coronary artery bypass graft surgery

CAC = coronary artery calcium

CHD = coronary heart disease

CI = confidence interval

CRP = C-reactive protein

FRS = Framingham Risk Score

JUPITER = Justification for the Use of Statins in Primary Prevention study

LDL-C = low-density lipoprotein in milligrams per deciliter

mSv = milisieverts

MI = myocardial infarction

PCI = percutaneous coronary intervention

QALYs = quality adjusted life years

RR = relative risk

It was assumed that every 1mg/dl decrease in LDL-C from statin therapy results in an absolute CHD risk reduction of 0.6% from each person’s initial CHD risk based on their baseline LDL-C, FRS or CAC score. [34]. Since statins result in a larger proportional LDL reduction in patients with a higher baseline LDL [31,34], in our model high-risk patients with high LDL receive a greater benefit of statin therapy (measured in relative risk for CHD event) compared to low-risk patients. Since women on average have a lower baseline LDL than men of the same age, women in our model were assigned a smaller relative reduction in risk from statin therapy than men. Additionally, since the low-risk population had significantly lower baseline LDL-C than the higher-risk population, we also modeled a decreased benefit of statin therapy in the low-risk compared to the high-risk patient population. In our basecase assumption we modeled a 19% decrease in statin efficacy in the low-risk compared to the high-risk population and through the variation of inputs in our PSA we modeled a decrease in statin efficacy in the low-risk compared to the high-risk population of as much as 60%. Additionally, while there is evidence of benefit from aspirin and statin therapy beyond CHD reduction, such as reduction in ischemic stroke, we only modeled a decrease in CHD risk for patients taking aspirin and statins [40,42].

In order to determine the adverse event rates and subsequent disutility associated with statin therapy we performed a meta-analysis using Stata/SE 13.0 (StataCorp, College Station, Texas) of all studies of high-dose atorvastatin that reported adverse event outcomes [49–53]. From our meta-analysis we derived expected event rates for myopathy, rhabdomyolysis, hepatitis, and liver failure (Table E in S1 File). Based on prior analyses we assumed that persons taking moderate-dose statins would experience roughly half of the above adverse events as compared to someone taking high-dose statins [18, 48]. Each adverse event carries with it a unique disutility and cost which are assigned in the model as decrements in QALYs and additional costs in US dollars. The disutilities and costs assigned to each adverse event were consistent with prior analyses of statin therapy. Costs associated with adverse events included not only the cost of initial work-up, laboratory testing, hospitalization and treatment but also the long-term costs of follow-up care and laboratory testing [18,47]. Given recent literature that demonstrated a significant increase in diabetes related to statin use, we modeled an increase incidence in diabetes as well as a decrement in quality of life associated with life-long diabetes for persons prescribed statins [7,54–56]. Lastly, since many of the adverse events on statin therapy reported by patients are subjective and related to muscle aches or symptoms unable to be quantified based on laboratory testing we modeled the incidence of ‘statin intolerance’ as well as a small quality of life decrement associated with subjective patient complaints from taking a statin [24,57,70]. While rates of reported muscle aches and statin intolerance vary greatly in randomized and observational studies of statin therapy, there appears to be a consistent increase in muscle aches in patients taking higher-intensity statins, and in particular atorvastatin [72–74]. Based on pooled estimates from multiple studies, we therefore assumed that 17.5% of patients on moderate and high-dose statins would be statin intolerant due to subjective symptoms [57, 70]. Since statin intolerance leads to statin discontinuation and typically manifests itself within the first six-months of statin therapy, we assumed a decreased basecase adherence to statin therapy in our model [57]. Thus, we used an adherence rate in the basecase model of 84%, selected to reflect both the reported adherence rates in clinical trials of statin therapy as well as the proportion of patients in observational studies having early intolerance to statin therapy [48,57]. The disutility and costs associated with aspirin therapy included potential gastrointestinal bleeding and hemorrhagic stroke, and life-long disutility was assumed for all cases of hemorrhagic stroke [40,42].

In order to validate our model outcomes with current data regarding the incidence of CHD, we correlated the predicted incidence of new CHD from our model with the most up-to-date statistics on CHD incidence in the US population based on calculating the actual rates of CHD from the US Heart Disease and Stroke statistics for men aged 45–75 and women aged 55–75 years old [72].

### Cost-Effectiveness Analysis

Over a 30-year time frame (2011–2040), future costs and QALYs were discounted by 3% annually [69]. Separately for men and women, incremental cost-effectiveness analyses were performed to compare all strategies against each other. Incremental cost-effectiveness ratios were calculated by dividing the difference in mean costs between two strategies by the difference in mean QALYs. An incremental-cost-effectiveness ratio (ICER) of $50,000 per QALY gained was used as a willingness-to-pay threshold to determine whether one strategy is determined to be cost-effective as compared to other less expensive but less effective strategies [59,75].

### Sensitivity Analyses

While the basecase model was based on a probabilistic sensitivity analysis, we performed several additional sensitivity analyses to assess the impact of key assumptions: the effects of aspirin use, the effect of varying adherence to medical therapy based on one knowing their CAC score as opposed to adherence rates demonstrated in the ‘real-world’ primary prevention patient population, varying radiation dosages from CAC tests, and the magnitude of disutility from statins. Regarding aspirin use for men, we assessed the following alternative assumptions: 1) aspirin was only prescribed for men with an FRS > 10% even in the treat-all arm, 2) in CAC-based interventions, aspirin was only prescribed when CAC > 0, and 3) aspirin was not prescribed in any interventions. Additionally, as there may be additional consequences of daily statin therapy not modeled in our basecase model including [76] decreased cognitive function [77], a more pronounced effect of statins on incidence diabetes, and the act of taking a pill, we varied the disutility associated with taking daily statins from 10 to 1,000 times the basecase disutility value. Since real-world statin adherence outside of the trial setting is typically lower, ranging from 19% to 52% [78–80], and since there is evidence that one’s knowledge of having a higher CAC score leads to improved adherence to statin therapy [81,82], we varied the assumed medication adherence from 19% to 52% for the non-CAC based strategies, and at the same time also assigned higher adherence rates to persons with elevated CAC scores (scores of zero, 1–99, 100–399, and >400 led to adherence rates of 36%, 52%, 56.5%, and 59% respectively) [82]. In the adherence based sensitivity analysis we assumed that each individual would undergo the respective assigned risk-stratification (CRP, CAC, and LDL) testing, recommended blood tests, as well as continue to follow-up with their physician regularly, but that their adherence to assigned medical therapy only would be decreased. We also performed a separate analysis in which we varied the radiation dose from a CAC test from 0.2 to 10 mSv which is the high range value for radiation dosage from CAC reported in the literature [28]. Since the ACC/AHA guidelines recommend that those with a CHD risk <7.5% by PCE not be started on high-dose statins, they leave open the approach for those with a PCE of 5–7.5% to be treated with moderate dose statins [7]. To better assess the approach to intermediate-low risk individuals in the ACC/AHA guidelines we performed a sensitivity analysis in which moderate-dose statins are prescribed for those with a PCE of 5–7.4% in the ACC/AHA strategy.

Additionally, subgroup analyses in which the costs of each strategy, as well as the number of individuals that would need to be included in a given strategy to prevent one CHD event (number needed to treat; NNT), and the number of individuals who would need to be included in a given strategy to lead to one additional strategy-related adverse event (number needed to harm; NNH) as compared to both the status quo and to ATP III simulations were performed. Subgroups evaluated included outcomes assessed for males and females separately as well as by age, with outcomes determined for men age 45–54, 55–64, and 65–74 years old and women age 55–64 and 65–74 years old separately. In order to assess outcomes in the low-risk population we performed additional sub-group analysis for men and women with an FRS <5%.

Lastly, in order to compare the results and global impact of our modeled strategies to other population wide interventions aimed at the primary prevention of CHD we compared the number of CHD events prevented, QALYs gained, and costs saved of the modeled ACC/AHA guidelines and a treat-all with high-dose statins approach to the following previously studied interventions: 1) Intensive behavior modification aimed at improving diet, increased exercise and physical activity, and weight loss which would lead to a 5% decrease in body mass index (BMI) nationally [83,84], 2) a national salt reduction program that would either lower salt intake by 1 or 3 grams per day [85], 3) a reduction in blood pressure using medication based interventions that would lead to the blood pressure of the population to be reduced to 135/75 based on the outcomes of the ALLHAT trial in which chlorthalidone, lisinopril, and amlodipine were used to lower systolic blood pressure (SBP) an average of 12 mm Hg and diastolic blood pressure (DBP) an average of 9 mm Hg in hypertensive individuals with at least one CHD risk factor [86,87], and 4) smoking cessation in which we assumed a one-time 1% decrease in smoking in the population nationally or an annual 1% reduction in smoking nationally for 30 years [88,89].

## Results

### Outcomes

**Fig 3:**
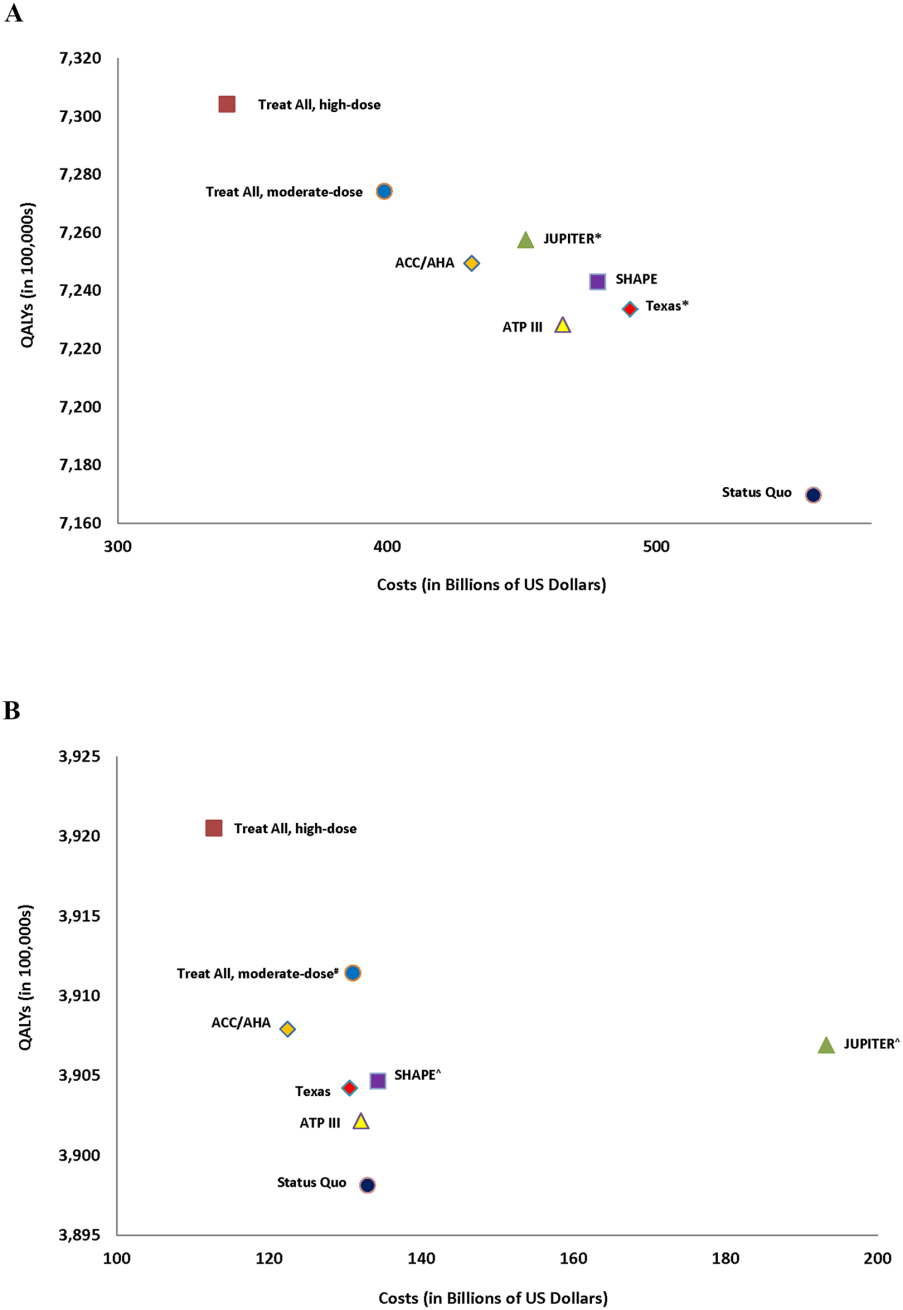
Results of basecase simulations. 3A: Men only. 3B: Women only. *Cost-effective as compared to less expensive AND less effective strategies with an incremental cost effectiveness ratio <$50,000/QALY gained for JUPITER compared to ACC/AHA and Texas compared to ATP III. #Cost-effective as compared to less expensive AND less effective strategies with an incremental cost effectiveness ratio <$50,000/QALY gained for treat-all with moderate-dose statins compared to ACC/AHA. ^Not cost-effective as compared to less expensive AND less effective strategies with an incremental cost effectiveness of ratio of > $50,000/QALY gained for both SHAPE and JUPITER compared to Texas. ICER = Incremental Cost Effectiveness Ratio. QALY = Quality-Adjusted Life Year. Other abbreviations same as in prior figures.

The basecase results are summarized in Table 3, Fig 3a and 3b, while strategy- and treatment-related complications are summarized in Table 4. All strategies for men were less costly and more effective than the status quo simulation, while for women all strategies except for JUPITER and SHAPE were less costly and more effective than the status quo simulation. Treat-all with high-dose statins was the most effective and least costly strategy. For men and women respectively, treat-all with high-dose statins gained more than 13.5 (95% CI 12.1–14.2) and 2.2 (95% CI 0.9–1.8) million QALYs, prevented 6.1 (95% CI 4.1–9.0) and 1.2 (95% CI 0.9–1.6) million MIs, and saved over $218 (95% CI $120-$346) and $20 (95% CI 0.6–35.1) billion, compared to the status quo simulation, despite leading to 12,149 (95% CI 650–35,469) bleeding complications, and causing an estimated 1,043,523 (95% CI 850,278–1,236,768) cases of hepatitis, 572,765 (95% CI 417,409–626,114) cases of myopathy, and 231,894 (95% CI 92,757–517,897) new diagnoses of diabetes. While one-third of statin-related adverse events in men were in the subgroup of the population at low-risk of CHD, more than 80% of statin-related adverse events in women were in the low-risk population. Treat-all with moderate-dose statins was more cost-effective than all risk-stratification approaches, dominating all other strategies for men and realizing an ICER of $24,362 per QALY gained as compared to ACC/AHA for women. The most cost-effective risk-stratification strategy was JUPITER followed by ACC/AHA for men, while ACC/AHA followed by Texas were the most cost-effective risk-stratification approaches for women. Texas led to only 26 million people undergoing CAC testing, but the SHAPE strategy led to almost 61 million CAC scans and resulted in 8,143 more radiation-attributable malignancies than the Texas strategy (Table 3). Lastly, ACC/AHA was less expensive and more effective than ATP III despite putting 15.2% more men and 5.0% more women, or a total of 8.7 million individuals, on high-dose statins.

**Table 3:** Outcomes for men and women based on microsimulations of one million patients for 2011–2040 ordered by increasing effectiveness (95% confidence interval). The cost effectiveness of each strategy is demonstrated as the cost per QALY gained as compared to the next less effective strategy.

**Men**						
	MIs prevented compared to ‘status quo’ (million)	% population on statins	CAC performed (millions)	Total QALYs gained compared to ‘status quo’ (millions)	Cost compared to status quo’ (billions $)	Cost effectiveness compared to next less effective strategy ($/QALY gained)
**ATP III**	2.5 (1.7, 4.5)	31.0% (28%, 34.2%)	—	+5.9 (4.9, 6.1)	-$93.1 (-$54.7,-$171.8)	$46,852 per QALY gained compared to status quo
**Texas**	2.1 (1.5, 4.0)	30.4% (27.7%, 33.5%)	24.5 (22.8, 26.0)	+6.4 (6.1, 6.8)	-$68.1 (-$35.1, -138.5)	$46,853 per QALY gained compared to ATP III*
**SHAPE**	2.7 (1.9, 4.5)	38.1% (35.1%, 41.1%)	47.0 (46.2, 47,6)	+7.3 (7.0, 7.8)	-$80.1 (-$40.5, -151.1)	Dominates^†^
**ACC/AHA**	3.4 (2.5, 4.9)	46.2% (42.4%, 49.4%)	—	+7.9 (6.8, 8.0)	-$126.9 (-$83.5,-$192.5)	Dominates^†^
**JUPITER**	3.6 (3.0, 5.1)	41.9% (38.7%, 44.9%)	—	+8.8 (7.8, 9.1)	-$106.9 (-$79.6,-$168.4)	$24,712 per QALY gained compared to ACC/AHA*
**Treat-all—Moderate-dose statins**	4.7 (2.8, 8.5)	100%	—	+10.5 (8.1, 11.7)	-$159.4 (-$65.1,-$310.4)	Dominates ^†^
**Treat-all—High-dose statins**	6.1 (4.1, 9.0)	100%	—	+13.5 (12.1, 14.2)	-$217.9 (-$120.0, -346.1)	Dominates^†^
**Women**						
	MIs prevented compared to ‘status quo’ (million)	% population on statins	CAC performed (millions)	Total QALYs gained compared to ‘status quo’ (millions)	Cost compared to status quo’ (billions $)	Cost effectiveness compared to next less effective strategy ($/QALY gained)
**ATP III**	0.20 (0.14, 0.26)	17.5% (15.0%, 20.0%)	—	+0.4 (+0.16, +0.6)	-$0.8 (+$1.5,-$3.1)	Dominates^†^
**Texas**	0.24 (0.22, 0.25)	17.1% (14.8%, 19.6%)	1.5 (1.1, 1.9)	+0.61 (+0.57, +0.65)	-$2.3 (-$1.0,-$4.0)	Dominates^†^
**SHAPE**	0.34 (0.32, 0.36)	24.9% (22.2%, 27.5%	13.8 (13.0, 14.8)	+0.65 (+0.52, +0.78)	+$1.8 (+$1.5, +$0.1)	$84,670 per QALY gained compared to Texas^#^
**JUPITER**	0.37 (0.30, 0.44)	53.1% (50.0%, 56.1%)	—	+0.88 (+0.56, +1.2)	+$60.3 (+65.6, +$55.0)	>$200,000 per QALY gained compared to SHAPE^#^
**ACC/AHA**	0.44 (0.35, 0.49)	21.5% (19.0%, 24.0%)	—	+1.0 (+0.74, +1.2)	-$10.5 (-$8.5,-$12.4)	Dominates^†^
**Treat-all—Moderate-dose statins**	0.70 (0.46, 1.0)	100%	—	+1.3 (+1.0, +1.65)	-$1.9 (+$15.2,-$19.1)	$23,362 per QALY gained compared to ACC/AHA*
**Treat-all—High-dose statins**	1.2 (0.87, 1.6)	100%	—	+1.4 (+1.0, +1.8)	-$20.2 (-$0.7,-$35.1)	Dominates^†^

*An incremental cost effectiveness ratio of < $50,000/QALY gained is considered cost effective

^#^ Not cost-effective as incremental cost-effectiveness ratio > $50,000/QALY gained

^†^Dominates denotes that the strategy is less expensive and more effective than the next less effective strategy

Abbreviations same as in prior table

**Table 4:** Rates of strategy- and treatment-related complications based on microsimulations for 2011–2040 for men and women.

	Radiation-attributable cancers	GI bleeds	Hemorrhagic strokes	Hepatitis without / with liver failure	Myopathy without / with rhabdomyolysis	Incident Diabetes
**ATP III**	0	3,383 (285, 9,915)	4,756 (402, 13,868)	273,737 (233,044, 324,429) / 1,237 (1,176, 1,317)	150,048 (125,716, 174,381) / 1,196 (0, 5,880)	60,830 (24,332, 135,855)
**Texas**	3,208	3,383 (285, 9,915)	4,756 (402, 13,868)	268,213 (218,544, 317,882) / 1,212 (1,152, 1,291)	147,020 (125,716, 174,381) / 1,712 (0, 5,762)	59,603 (23,841, 135,855)
**SHAPE**	11,351	3,383 (285, 9,915)	4,756 (402, 13,868)	348,933 (284,516, 413,550) / 1,577 (1,499, 1,680)	191,267 (160,251, 222,283) / 1,525 (0, 7,496)	77,541 (31,016, 173,174)
**JUPITER**	0	3,383 (285, 9915)	4,756 (402, 13,868)	478,514 (389,901, 567,128) / 2,612 (2,055, 2,304)	262,297 (219,762, 304,831) / 2,091 (0, 10,279)	106,336 (42,535, 237,485)
**ACC/AHA**	0	3,383 (285, 9915)	4,756 (402, 13,868)	391,074 (318,653, 463,495) / 1,767 (1,680, 1,883)	214,367 (179,064, 249,128) / 1,709 (0, 8,401)	86,905 (34,762, 194,089)
**Treat-all—Moderate-dose Statins**	0	5,050 (425, 14,799)	7,099 (600, 20,670)	521,761 (417,409, 626,114) / 2,358 (1,469, 2,512)	286,003 (239,624, 332,381) / 2,280 (0, 11,208)	77,298 (30,919, 172,632)
**Treat-all—High-dose statins**	0	5,050 (425, 14,799)	7,099 (600, 20,670)	1,043,523 (850,278, 1,236,768) / 4,715 (4,483, 5,024)	572,005 (479,248, 664,763) / 4,561 (0, 22,416)	231,894 (92,757, 517,897)

GI = gastrointestinal

Abbreviations same as in prior tables

**Fig 4:**
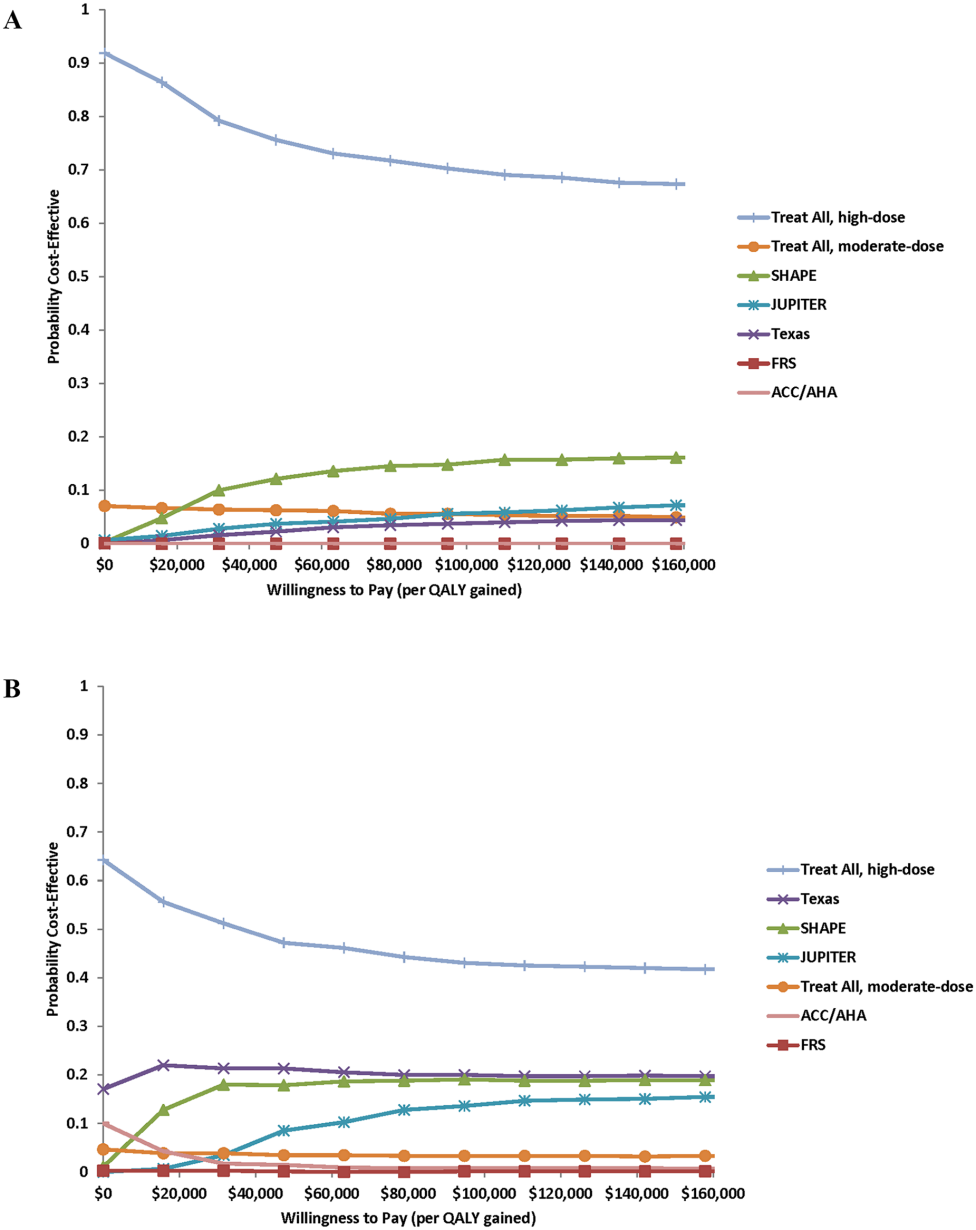
Acceptability curves for basecase simulations. 4a: Men only. 4b: Women only. Abbreviations same as in prior figures.

Regardless of the willingness-to-pay threshold used, (Fig 4a and 4b), treat-all with high-dose statins dominated all other strategies for both men and women. While for men there was >80% certainty that treat-all with high-dose statins is the most cost-effective approach at a willingness-to-pay threshold of $50,000 per QALY gained, for women there was greater uncertainty in the cost-effectiveness of treat-all with high-dose statins as at a willingness-to-pay threshold of $50,000 per QALY gained there was less than a 50% chance that treat-all with high-dose statins was the most cost-effective approach.

Our model results for incidence of CHD for the status quo simulation were markedly similar to the actual real incidence of CHD in our studied population. The most recent US national statistics from the American Heart Association demonstrated 10.3 million new cases of CHD in males age 45 to 75 and 3.9 million in females age 55 to 75, while our model predicted that there would be 12.7 million cases of new CHD in males and 3.3 million cases of new CHD in females in the same age ranges.

### Sensitivity Analyses

**Fig 5:**
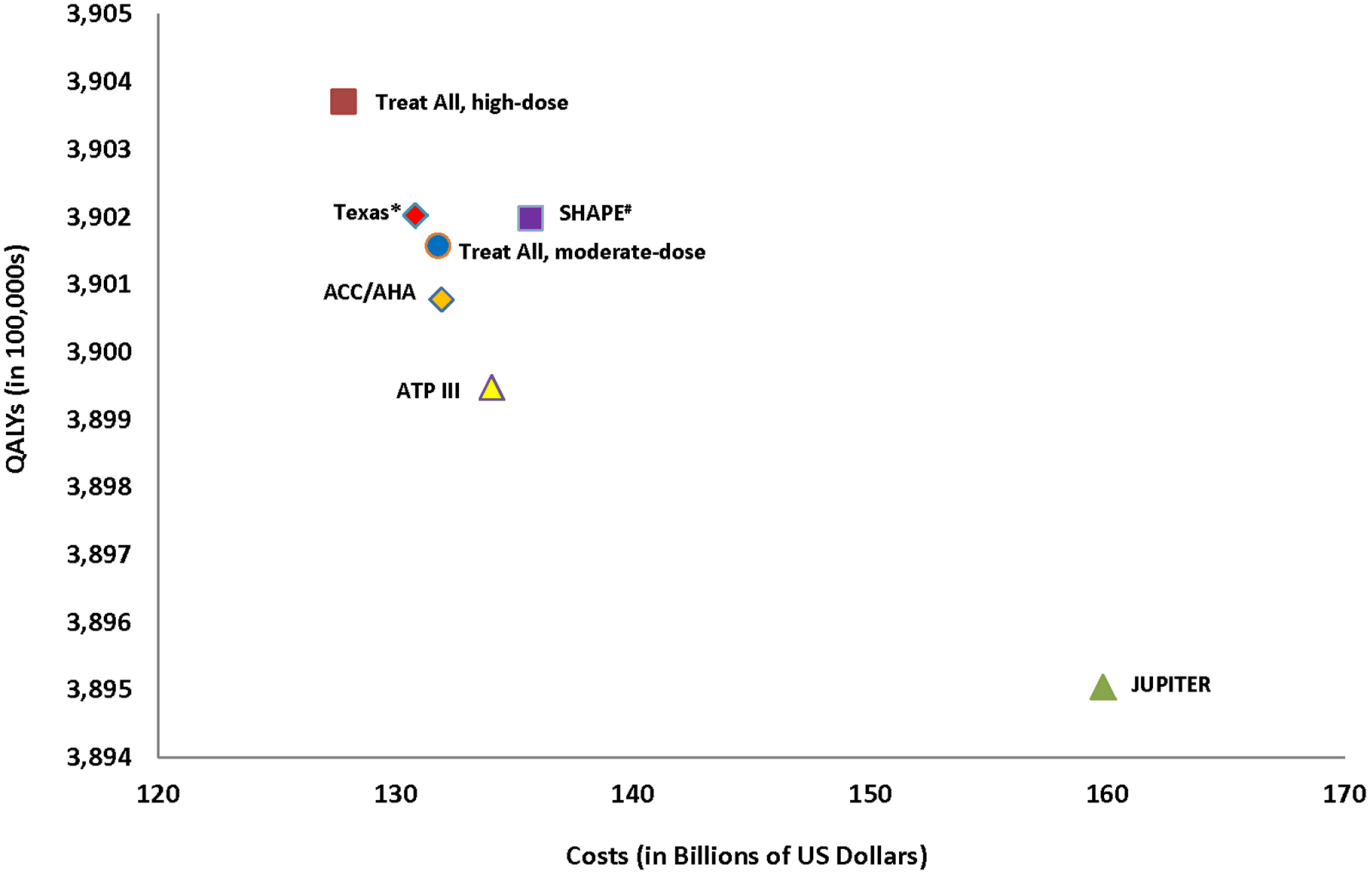
Adherence Sensitivity analysis: Cost-effectiveness of strategies evaluated using an adherence rate of 19% for all non-CAC based strategies and a variable adherence rate for CAC based strategies in which the higher the CAC score the higher the adherence to therapy (women only simulation shown). *Texas dominates treat-all with moderate-dose statins as well as all other risk-stratification strategies. #SHAPE is not cost-effective as compared to treat-all with moderate-dose statins as the ICER of SHAPE compared to treat-all with moderate-dose statins is $95,864 per QALY gained. Abbreviations same as in prior figures.

**Fig 6:**
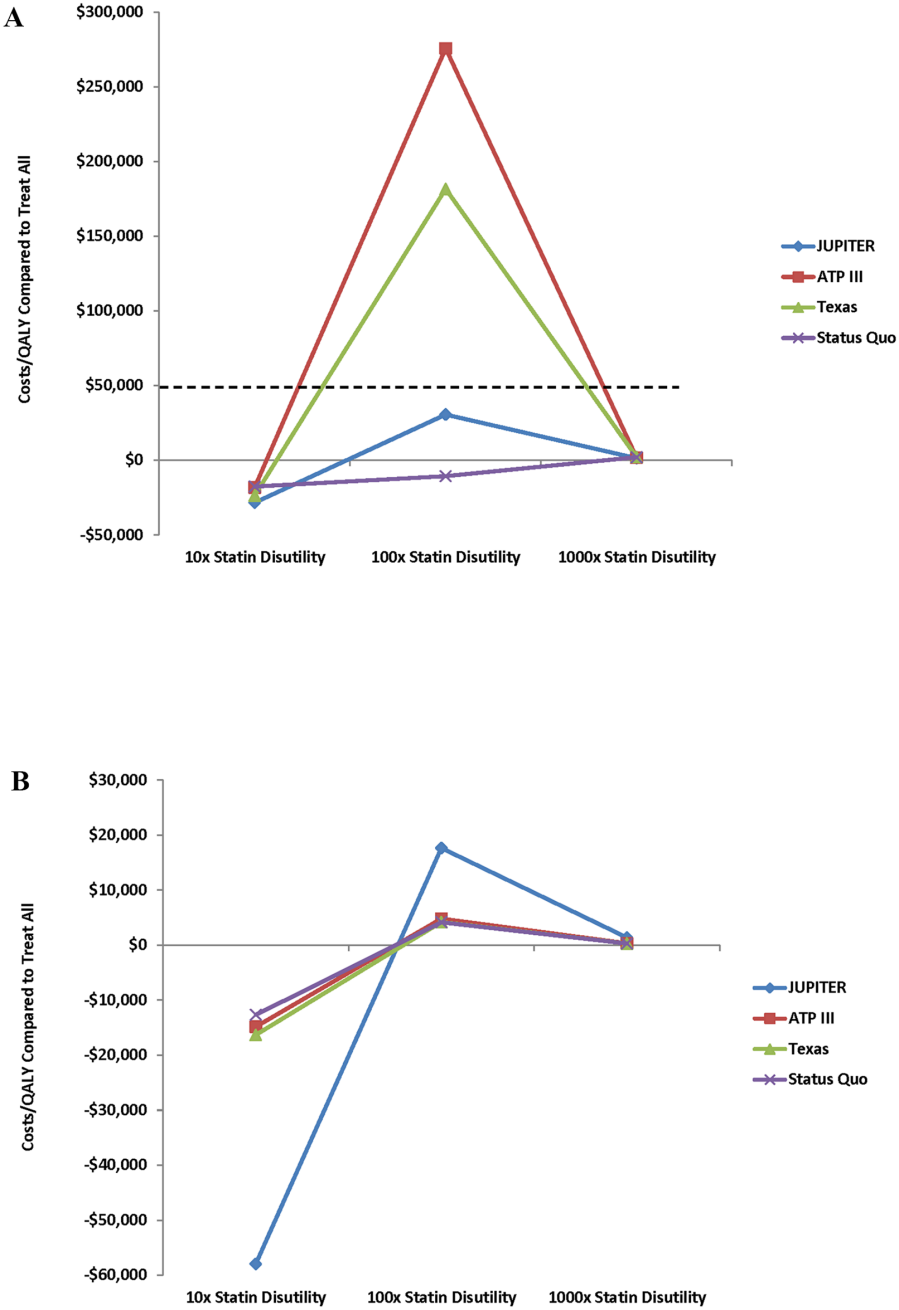
Sensitivity Analysis: Costs per QALY of selected* strategies compared to treat-all with high-dose statins as a function of increasing statin disutility for men and women^#^. 6a: Men (cost-effectiveness threshold of $50,000 per QALY gained demonstrated by the dashed line). 6b: Women. *Only strategies that were not dominated by treat-all with high-dose statins at 100-times basecase statin disutility are shown. #At 10-times the basecase statin disutility all strategies are more expensive and less costly than treat-all with high-dose statins, therefore they are dominated by treat-all high-dose statins and demonstrate a negative cost per QALY. At 100-times basecase statin disutility for men, status quo remains dominated by treat-all with high-dose statins and therefore retains a negative cost per QALY, while the other strategies shown are now more effective than treat-all with high-dose statins but more expensive, evidenced by a positive cost per QALY. Only JUPITER is cost-effective as compared to treat-all with high-dose statins as the cost per QALY gained via the JUPITER strategy is < $50,000 (noted by the dashed line) as compared to treat-all with high-dose statins. At 100-times basecase statin disutility for women and 1,000-times statin disutility for both men and women all strategies shown are more effective and more expensive than treat-all with high-dose statins but they are so much more effective than treat-all with high-dose statins that the cost per QALY gained is low and they are all more cost effective compared to treat-all with high-dose statins. Abbreviations same as in prior figures.

For men, regardless of the adherence rate for non-CAC based strategies tested, even as low as 19%, treat-all with high-dose statins dominated all other strategies and treat-all with moderate-dose statins dominated all risk-stratification based approaches (Table F in S1 File). For women, even at an adherence rate of 19% for all non-CAC based strategies, treat-all with high-dose statins still remained the dominant strategy, however Texas becomes dominant over treat-all with moderate-dose statins (Fig 5 and Table F in S1 File). As statin disutility is progressively increased, treat-all with high-dose statins becomes less cost-effective. For both men and women when the disutility from statin therapy is only increased by a factor of 10, treat-all with high-dose statins remains the dominant strategy. However, at 100 times the basecase statin disutility, treat-all with high-dose statins remains the cheapest strategy in men, but JUPITER becomes more cost-effective with an ICER of $30,623 per QALY gained compared to treat-all high dose, while for women essentially all risk-stratification approaches become more cost effective than treat-all with high-dose statins, but the status quo simulation is actually the most effective approach with an ICER of only $4,154 per QALY gained compared to treat-all with high-dose statins. By 1,000 times the basecase statin disutility, the status quo approach is more cost-effective for both men and women (Fig 6a and 6b, Table G in S1 File). Additionally, at 100 times the basecase statin disutility, ATP III becomes more cost-effective than the ACC/AHA approach. At lower adherence rates and when assigned an increased disutility rate to statin therapy, the 95% CIs of the QALYs gained and costs saved of the treat-all with high-dose statins and other risk stratification approaches begin to cross, indicating that the cost-effectiveness of treat-all is more uncertain as the adherence rate to therapy decreases and the disutility from statin therapy increases (Fig 6a and 6b, Table G in S1 File). While some studies have demonstrated an increased risk of hemorrhagic stroke with statins, even when we assumed a relative risk of hemorrhagic stroke with statin therapy of between 1.15 and 1.38 [34], treat-all with high-dose statins remained the most cost-effective approach despite the fact that the NNH with statin therapy decreased in this simulation.

Removing aspirin from the model or only assigning aspirin to those with an FRS>10% for all strategies led to JUPITER in men and Texas in women being more cost effective than treat-all with moderate-dose statins, however treat-all with high-dose statins remained the dominant strategy in all aspirin-related sensitivity analyses (Table H in S1 File). Additionally, varying the radiation dose for a CAC test did not change the overall order of cost-effectiveness for each of the simulated interventions (Table I in S1 File). Lastly, broadening the ACC/AHA guideline recommendations to treat all individuals with a PCE of 5–7.4% with moderate-dose statins did not change the basecase hierarchy of primary prevention strategies for either men or women but did lead to 54.5% of men and 26.6% of women being placed on statin therapy, an increase of 9% and 5% in statin use as compared to the basecase ACC/AHA strategy (Table J in S1 File).

While treat-all with high-dose statins remains the most cost-effective approach regardless of age, gender, or FRS of the population evaluated, in younger, lower-risk, and female populations the NNH decreases and the NNT increases for all interventions (Tables 5 and 6). In particular, in the low-risk female population (FRS <5%) the NNH for the treat-all population as compared to either status quo or ATP III is actually lower than the NNT, indicating that while treat-all with high-dose statins is effective at reducing CHD events in this population, it does so at the expense of putting individuals at a higher risk of statin-related adverse events (Table 6). Additionally, at a younger age and at lower risk, ACC/AHA becomes less cost-effective while JUPITER becomes a more cost-effective risk stratification strategy (Tables 5 and 6).

**Table 5:** Age and Gender Sub-group Analysis: Outcomes for each strategy evaluated based on specific age and gender sub-group analyses—including number needed to treat9* and number needed to harm^#^ for each strategy as compared to status quo and ATP III (strategies listed in order of increasing effectiveness).

**All Men**						
	Number needed to treat to prevent one CHD event as compared to status quo	Number needed to harm to cause one additional strategy-related adverse event as compared to status quo	Number needed to treat to prevent one CHD event as compared to ATP III	Number needed to harm to cause one additional strategy-related adverse event as compared to ATP III	% population on statins	Total Cost(billions $)
**ATP III**	20 (11, 29)	207 (178, 242)	**—**	**—**	31.0% (28%, 34.2%)	$425 ($325, $565)
**Texas**	23.5 (12, 33)	212 (183, 256)	ATP III fewer CHD events^^^	ATP III more adverse events^†^	30.4% (27.7%, 33.5%)	$490 ($346, $584)
**SHAPE**	18.5 (11, 26)	151 (137, 171)	250 (236, 1000)	579 (408, 1,000)	38.1% (35.1%, 41.1%)	$478 ($368, $579)
**ACC/AHA**	15 (10, 20)	107 (117, 129)	62.5 (56, 111)	272 (224, 347)	46.2% (42.4%, 49.4%)	$431.5 ($305, $536)
**JUPITER**	14 (10, 16)	134 (122, 149)	37 (44.5, 77)	380 (297, 536)	41.9% (38.7%, 44.9%)	$451.5 ($329, $540)
**Treat-all—Moderate-dose stains**	10.5 (6, 18)	112 (82, 159)	23 (12.5, 45.5)	239 (147, 704)	100%	$399 ($187, $554)
**Treat-all—High-dose statins**	8 (5.5, 12)	46 (34, 64)	14 (11, 21)	60 (41, 93)	100%	$340.5 ($151, $499)
**All Women**						
	Number needed to treat to prevent one CHD event as compared to status quo	Number needed to harm to cause one additional strategy-related adverse event as compared to status quo	Number needed to treat to prevent one CHD event as compared to ATP III	Number needed to harm to cause one additional strategy-related adverse event as compared to ATP III	% population on statins	Total Cost(billions $)
**ATP III**	143 (139, 200)	683 (481, 1,153)	**—**	**—**	17.5% (15.0%, 20.0%)	$150 ($122, 181)
**Texas**	114 (111, 125)	723 (504, 1,217)	618 (250, 1000)	ATP III more adverse events†	17.1% (14.8%, 19.6%)	$149 ($124, $175
**SHAPE**	79 (77, 83)	306 (257, 383)	167 (143, 181)	554 (411, 870)	24.9% (22.2%, 27.5%	$153 ($127, $181)
**JUPITER**	73 (62.5, 91)	99 (92, 107)	154 (111, 167)	116 (107, 127)	53.1% (50.0%, 56.1%)	$220 ($188, $254)
**ACC/AHA**	62 (55.5, 77)	410 (328, 544)	111 (91, 125)	1,023 (632, 2,687)	21.5% (19.0%, 24.0%)	$139 ($111, $169)
**Treat-all—Moderate-dose stains**	39 (27, 59)	114 (83, 162)	54.5 (33, 83)	137 (95, 213)	100%	$149 ($104, $196)
**Treat-all—High-dose statins**	23 (17.5, 31)	47 (34, 65)	28 (20, 37)	50 (36, 71)	100%	$128 ($65, $178)
**Men Age 45–54**						
	Number needed to treat to prevent one CHD event as compared to status quo	Number needed to harm to cause one additional strategy-related adverse event as compared to status quo	Number needed to treat to prevent one CHD event as compared to ATP III	Number needed to harm to cause one additional strategy-related adverse event as compared to ATP III	% population on statins	Total Cost(billions $)
**ATP III**	30.5 (21, 45.5)	245 (219, 298)	**—**	**—**	21.6% (19.3, 24.3%)	$231 ($189, $269)
**Texas**	25 (21, 30)	317 (267,390)	940 (91, 1000)	ATP III more adverse events†	18.4% (16%, 20.9%)	$220 ($185, $253)
**ACC/AHA**	20 (16, 27)	157 (141, 175)	61 (55, 67)	408 (317, 562)	31.8% (29.0%, 34.7%)	$217 ($175, $256)
**SHAPE**	21 (17, 26)	198 (174, 227)	64 (59, 91)	893 (546, 2,096)	26.2% (23.6%, 29.2%)	$218 ($182, $251)
**JUPITER**	19.5 (15, 23)	229 (202, 267)	50 (45.5, 54.5)	2,263 (973, 5,502)	23.5% (20.9%, 25.9%)	$214 ($170, $248)
**Treat-all—Moderate-dose stains**	10 (6, 18.5)	98 (74, 131)	15.5 (8, 31)	160 (105, 272)	100%	$188 ($86, $263)
**Treat-all—High-dose statins**	8 (5.5, 12)	44 (33, 59)	11 (7, 16)	53 (37, 77)	100%	$160 ($75, $233)
**Men Age 55–64**						
	Number needed to treat to prevent one CHD event as compared to status quo	Number needed to harm to cause one additional strategy-related adverse event as compared to status quo	Number needed to treat to prevent one CHD event as compared to ATP III	Number needed to harm to cause one additional strategy-related adverse event as compared to ATP III	% population on statins	Total Cost(billions $)
**Texas**	18 (10, 32)	212 (185, 250)	ATP III fewer CHD events^	ATP III more adverse events†	33.7% (30.7, 36.5%)	$171 ($120, $208)
**ATP III**	16 (9, 26)	193 (169, 226)	**—**	**—**	35,7% (32.6%, 38.8%)	$164 ($104, $205)
**SHAPE**	14 (9, 23)	139 (125, 155)	200 (145, 1,000)	489 (354, 786)	44.0% (40.8%, 47.2%)	$166 ($114, $207)
**JUPITER**	11.5 (8, 16)	124 (114, 137)	43 (41, 71)	344 (275, 474)	47.7% (44.4%, 50.7%)	$160 ($108, $201)
**ACC/AHA**	11 (8, 16)	73 (70, 77)	42 (34, 59)	118 (109, 127)	70.6% (68.1%, 73.6%)	$147 ($98, $194)
**Treat-all—Moderate-dose stains**	10 (6, 18.5)	124 (88, 183)	27 (16, 62)	348 (162, 3,527)	100%	$145 ($67, $204)
**Treat-all—High-dose statins**	8 (5.5, 12)	48 (35, 68)	16 (14, 22)	64 (43, 104)	100%	$124 ($59, $184)
**Men Age 65–74**						
	Number needed to treat to prevent one CHD event as compared to status quo	Number needed to harm to cause one additional strategy-related adverse event as compared to status quo	Number needed to treat to prevent one CHD event as compared to ATP III	Number needed to harm to cause one additional strategy-related adverse event as compared to ATP III	% population on statins	Total Cost(billions $)
**Texas**	28 (10, 500)	128 (118, 143)	ATP III fewer CHD events^	544 (386, 918)	49.8% (46.7, 52.9%)	$90 ($54, $116)
**SHAPE**	20 (9, 77)	117 (107, 128)	ATP III fewer CHD events^	382 (295, 537)	53.0% (49.9%, 56.2%)	$855 ($504, $1,128)
**ATP III**	15 (8, 27)	169 (150, 195)	**—**	**—**	42.4% (39.1%, 45.4%)	$65 ($32, $88)
**Treat-all—Moderate-dose statins**	12 (7, 22)	139 (96, 218)	66 (58, 125)	221 (99, 746)	100%	$62 ($30, $87)
**ACC/AHA**	11 (7, 17)	72 (69, 75)	43 (45, 62)	126 (116, 137)	75.1% (72.5%, 78.8%)	$57 ($29, $82)
**JUPITER**	11 (7, 14)	80 (76, 85)	36 (29, 67)	152 (138, 170)	69.6% (66.6%, 72.3%)	$72 ($46, $93)
**Treat-all—High-dose statins**	9.5 (6.5, 14)	50 (36, 72)	26 (30, 33)	72 (46, 125)	100%	$52 ($24, $78)
**Women Age 55–64**						
	Number needed to treat to prevent one CHD event as compared to status quo	Number needed to harm to cause one additional strategy-related adverse event as compared to status quo	Number needed to treat to prevent one CHD event as compared to ATP III	Number needed to harm to cause one additional strategy-related adverse event as compared to ATP III	% population on statins	Total Cost(billions $)
**ATP III**	151 (143, 160)	504 (392, 694)	**—**	**—**	17.0% (14.8%, 19.4%)	$90 ($74, 107)
**Texas**	131 (100, 156)	524 (399, 730)	948 (333, 1000)	ATP III more adverse events†	16.7% (14.5%, 19.2%)	$88 ($73, $105)
**SHAPE**	105 (91, 119)	404 (324, 526)	333 (250, 339)	2,037 (905, 12,318)	19.1% (16.7%, 21.6%)	$89 ($73, $105.5)
**ACC/AHA**	75 (71, 77)	422 (337, 586)	150 (143, 169)	2,577 (1.018, 3,619)	18.6% (15.9%, 21.1%)	$84 ($69, $101)
**JUPITER**	51 (48, 52)	73 (69, 77)	77 (71, 83)	85 (80, 91)	65.5% (62.7%, 68.6%)	$145 ($124, $168)
**Treat-all—Moderate-dose stains**	39 (29, 45.5)	106 (79, 148)	52 (37, 69)	135 (94, 208)	100%	$89 ($64, $116)
**Treat-all—High-dose statins**	23 (18, 27)	45 (34, 62)	28 (20, 33)	50 (36, 71)	100%	$77 ($48, $107)
**Women Age 65–74**						
	Number needed to treat to prevent one CHD event as compared to status quo	Number needed to harm to cause one additional strategy-related adverse event as compared to status quo	Number needed to treat to prevent one CHD event as compared to ATP III	Number needed to harm to cause one additional strategy-related adverse event as compared to ATP III	% population on statins	Total Cost(billions $)
**JUPITER**	203 (125, 278)	247 (210, 298)	ATP III fewer CHD events^	271 (228, 335)	32.3% (29.5%, 35.3%)	69 (58, 81)
**ATP III**	119 (114, 125)	2,694) (1,045,4,873)	**—**	**—**	17.2% (14.8%, 19.6%)	56 (47, 66)
**Texas**	96 (83, 100)	3,649 (1,222, 4,057)	494 (250, 603)	ATP III more adverse events†	16.8% (14.6%, 19.0%)	56 (46, 66)
**SHAPE**	55 (48, 62.5)	242 (209, 289)	104 (77, 125)	266 (226, 324)	32.7% (29.9%, 35.4%)	59 (48, 71)
**Treat-all—Moderate-dose stains**	37 (26, 48)	129 (91, 195)	54 (32, 77)	136 (94, 210)	100%	54 (38, 69)
**ACC/AHA**	38 (28, 48)	211 (182, 247)	56 (37, 77)	229 (196, 271)	35.2% (32.4%, 38.3%)	50 (37, 61)
**Treat-all—High-dose statins**	22 (16, 28)	49 (36, 69)	28 (18, 36)	50 (36, 71)	100%	46 (28, 63)

*The number needed to treat (NNT) is based on the number of people that need to be included in a given primary prevention strategy to prevent one CHD event as compared to either status quo or ATP III.

^#^The number needed to harm (NNH) represents the number of people that would need to be included in a given primary prevention strategy in order for that strategy to lead to one more additional strategy-related adverse event (which include statin induced myopathy, rhabdomyolysis, hepatitis, liver failure, incident diabetes, and radiation associated malignancy from diagnostic cardiac imaging) as compared to either status quo or ATP III.

^^^Unable to calculate number needed to treat to allow this strategy to be more effective than ATP III, since ATP III led to fewer CHD events compared to the strategy being evaluated

^†^Unable to calculate the number needed to harm to allow ATP III to be more effective than this strategy, since ATP III led to more medication related adverse events compared to the strategy being evaluated

**Table 6:** Low-risk Subgroup Analysis: Outcomes for each strategy evaluated for men and women with a Framingham Risk Score (FRS) of less than 5%—including the number needed to treat and the number needed to harm for each strategy as compared to status quo and ATP III (strategies listed in order of increasing effectiveness)* ^#^.

**Low-risk Men**						
	Number needed to treat to prevent one CHD event as compared to status quo	Number needed to harm to cause one additional strategy-related adverse event as compared to status quo	Number needed to treat to prevent one CHD event as compared to ATP III	Number needed to harm to cause one additional strategy-related adverse event as compared to ATP III	% population on statins	Total Cost(billions $)
**ACC/AHA**	468 (331, 1,004)	1,296 (889, 2,235)	ATP III fewer CHD events^^^	ATP III more adverse events^†^	6.0% (4.7%, 7.5%)	$29.5 ($23, $36)
**SHAPE**	217 (167, 333)	239 (204, 272)	665 (299, 1,054)	512 (388, 745)	20.5% (18.0%, 23.1%)	$32 ($26, $39)
**ATP III**	321 (326, 495)	430 (355, 540)	**—**	**—**	12.4% (10.5%, 14.5%)	$30 ($24, $37)
**JUPITER**	36 (32, 42)	430 (355, 540)	40 (36, 45)	ATP III more adverse events^†^	12.9% (10.7%, 14.8%)	$21 ($16, $26)
**Treat-all—Moderate-dose statins**	32 (21, 45)	89 (69, 115)	35 (22, 53)	117 (85, 169)	100%	$28 ($15, $42)
**Treat-all—High-dose statins**	25 (20, 31)	42 (32, 56)	28 (21, 34)	47 (35, 66)	100%	$25 ($13, $38)
**Low-risk Women**						
	Number needed to treat to prevent one CHD event as compared to status quo	Number needed to harm to cause one additional strategy-related adverse event as compared to status quo	Number needed to treat to prevent one CHD event as compared to ATP III	Number needed to harm to cause one additional strategy-related adverse event as compared to ATP III	% population on statins	Total Cost(billions $)
**SHAPE**	253 (200, 268)	233 (202, 274)	598 (500, 988)	358 (289, 466)	22.3% (19.6%, 25.0%)	$66) ($51, $82)
**ATP III**	437 (331, 502)	665 (505, 922)	**—**	**—**	10.7% (9.0%, 12.7%)	$58 ($45, $73)
**ACC/AHA**	228 (212, 245)	373 (309, 476)	477 (397, 1,000)	849 (577, 1,676)	15.6% (13.2%, 17.9%)	$57 ($44, $72)
**JUPITER**	147 (125, 167)	665 (505, 922)	222 (200, 333)	ATP III more adverse events^†^	11.4% (9.6%, 13.3%)	$55 ($42, $68)
**Treat-all—Moderate-dose statins**	75 (63, 83)	96 (73, 128)	91 (77, 111)	112 (82, 158)	100%	$65 ($46, $93)
**Treat-all—High-dose statins**	46 (40, 53)	43 (33, 58)	51 (45, 63)	46 (34, 64)	100%	$58 ($38, $85)

*The number needed to treat (NNT) is based on the number of people that need to be included in a given primary prevention strategy to prevent one CHD event as compared to either status quo or ATP III, while the number needed to harm (NNH) represents the number of people that would need to be included in a given primary prevention strategy in order for that strategy to lead to one more additional strategy-related adverse event as compared to either status quo or ATP III.

^#^Texas not shown as at Framingham Risk (FRS) of < 10% ATP III and Texas become identical strategies since Texas only recommends coronary artery calcium scans for people with an FRS of > 10%.

^^^Unable to calculate number needed to treat to allow this strategy to be more effective than ATP III, since ATP III led to fewer CHD events compared to the strategy being evaluated

^†^Unable to calculate the number needed to harm to allow ATP III to be more effective than this strategy, since ATP III led to more medication related adverse events compared to the strategy being evaluated

Lastly, in comparison to the modeled ACC/AHA guideline and treat-all with high-dose statin interventions, sustained high impact interventions such as large scale salt reduction in the population and continued and cumulative smoking cessation over a long time period can lead to a greater reduction in CHD events and QALYs gained compared to the ACC/AHA guideline implementation, but they do not realize the same benefit in CHD reduction and quality of life as compared to treating the entire at-risk population with high-dose statins (Table 7). Weight loss will initially lead to a decrease in CHD risk but over time individuals tend to re-gain a proportion of their weight, making intense diet and behavior modification strategies less effective long term as compared to population based strategies to reduce salt intake, decrease, smoking, or prescribe statins judiciously to those at-risk. While treating hypertensive patients with medical therapy can lead to a greater benefit in outcomes and quality of life compared to global salt reduction, the medication based approach to reducing blood pressure adds costs to society. Moreover, since the global reduction in risk via sustained smoking cessation and large scale salt reduction can save more money than treating all individuals with high-dose statins, even though these interventions lead to a smaller improvement in quality of life as compared to treat-all, for a given QALY gained they would likely be less expensive strategies as compared to treat-all with high-dose statins.

**Table 7:** Comparison of national implementation of the 2013 ACC/AHA guidelines and treating all persons with high-dose statins strategies with other population based strategies—Comparing CHD events prevented, costs saved, and QALYs gained for each strategy compared to status quo.

Intervention	New CHD events prevented (millions)	Costs Saved (billions)	QALYs realized (millions)	Reference
Modeled Strategies				
ACC/AHA 2013 Guidelines	3.9	$137	8.9	Galper et al. (this manuscript)
Treat-all with High-dose Statins	7.3	$237	14.9	Galper et al. (this manuscript)
Comparable Population Strategies				
5% decrease in BMI*	1.8	-$6.2 (strategy costs more than status quo)	0.50	[79,80]
Salt reduction by 3 gm/day	1.8 to 3.6	$354 to $603	6.6 to 10.5	[81]
Salt reduction by 1gm/day	0.66 to 1.1	$114 to $201	2.25 to 3.6	[81]
Reduce BP to 135/75 using medication interventions from ALLHAT study^#^	3.0	-$159 (strategy costs more than status quo)	10.8	[82,83]
One-time 1% decrease in smoking	.093	$8.6	0.17	[84,85]
Annual 1% decrease in smoking	6.9	$430	12.0	[84,85]

*Costs and QALYs of weight reduction based on lifetime model of intense dietary, exercise, and lifestyle modification which assumed that over time participants would gain back a significant component of their initial weight loss.

^#^Based on average blood pressure reduction from all medication interventions in ALLHAT which included chlorthalidone, amlodipine, and Lisinopril for hypertensive patients with at least one CHD risk factor. The blood pressure reduction achieved was a decrease in SBP of 12mm Hg and in DBP of 9mm Hg. At studies end 66% of patients achieved a blood pressure of goal of < 140/90

ALLHAT = The antihypertensive and lipid lowering treatment to prevent heart attack trial, BMI = body mass index, CHD = coronary heart disease, DBP = diastolic blood pressure, QALY = quality adjusted life year, SBP = systolic blood pressure

## Discussion

### Treating All Compared to Risk-Stratification

Our results suggest that for both men and women, treating all individuals with high-dose generic statins and all men with low-dose aspirin may be more effective and less costly than risk-stratification approaches such as SHAPE, the Texas Heart Attack Prevention Law, the JUPITER approach, ATP III, and the ACC/AHA lipid guidelines. Our study builds on prior research which demonstrated that treating a person based on overall risk and not a specific LDL-C target, or utilizing a treat-all approach for certain patient populations as compared to individual risk-stratification approaches, is a cost-effective approach to primary prevention [6,18,19].While previous analyses have evaluated individual approaches to improving risk-stratification and primary prevention of CHD in asymptomatic intermediate- to high-risk persons [6,22–24,90,91], we believe that the present study is the first comprehensive cost-effectiveness analysis of all major approaches to risk-stratification and prevention of CHD. Additionally, with the advent of low-cost generic statins and the evidence showing their safety, it may actually be feasible and more cost-effective to treat individuals over a certain age threshold rather than stratifying future CHD risk by LDL-C, FRS, PCE, CAC, or CRP levels [18,19]. While our findings are consistent with another recently published analysis which demonstrated that treating all individuals with generic priced statins is a cost-effective approach as compared to utilizing a CAC score based approach to the primary prevention of CHD [91], our model builds further on this analysis by including multiple approaches to risk stratification as compared to a treat-all approach and by including robust sensitivity analyses to demonstrate the effect of varying the adherence to therapy, assigned disutility associated with statin therapy, and the risk of future CHD in the treated population on the cost-effectiveness of primary prevention strategies.

While the broad use of generic statins appears to be cost-effective, there is active debate regarding whether treating all intermediate-risk persons with generic statins is medically justifiable [20,92]. Despite the fact that treat-all placed many more people on statins and led to a modest increase in medication related side effects, including over 200,000 new cases of diabetes, it also prevented more MIs and led to more life-years and QALYs gained. In comparison, risk-stratification-based approaches identify individuals with greater risks of developing CHD based on an elevated FRS, PCE, CAC, or CRP level, but they may not sufficiently lower risks across the entire population. For example, of the estimated 300,000 individuals dying of sudden cardiac death each year in the United States, a clear majority do not have previously established CHD or even high-risk profiles [93]; while risk-stratifying approaches might help to identify those people who are at very low risk, they may fail to identify many of these individuals whose CHD risk is not adequately captured through risk stratification and who could thus potentially benefit from statin therapy. However, our analysis also demonstrates that for low-risk individuals, particularly women, the decreased risk of CHD events realized through a treat-all with high-dose statins approach may be tempered by an increased risk of statin-related adverse events. Additionally, while there is relatively little uncertainty that treat-all with high-dose statins is the most cost-effective strategy for men based on our simulations, the likelihood that treat-all with high-dose statins is the most cost-effective approach for women is less than 50%, and while the 95% CIs for QALYs gained for treat-all with high-dose statins and other risk stratification approaches do not cross for men they do overlap for JUPITER and ACC/AHA for women, indicating significant uncertainty regarding the optimal approach to primary prevention in women. Our analysis also demonstrated that if treat-all is deemed unfeasible as a clinical policy then utilizing a risk-stratification approach based on JUPITER for men and ACC/AHA guidelines or a combined risk factors/CAC approach, as in the Texas legislation, for women, could be reasonably cost-effective alternatives for primary prevention. Moreover, our analysis demonstrates that when considering the use of CAC to risk stratify patients for the primary prevention of CHD, perhaps a hybrid approach similar to what has been proposed in the Texas legislation, in which only those with an elevated risk of CHD undergo CAC testing, may be more a cost-effective approach instead of recommending that most of the population evaluated undergo CAC testing as in SHAPE. Additionally, while our model was based on a United States population, the inputs that drove the model were based on large international randomized trials and we also correlated FRS to PCE which was developed utilizing data regarding ASCVD risk of populations from multiple ethnic backgrounds. It is possible that, depending on the costs of interventions in non-US populations, utilizing a treat-all with high-dose statins approach in the appropriate patient population may also be a cost-effective alternative for the primary prevention of CHD in an international setting.

### Addressing Statin Disutility

Our model assumed rates of hepatitis and myopathy related to statin use that were even higher than those in recently-published meta-analyses [18,76], and while statins appear to lead to a low but observed increase in risk of new diabetes [54,76], our analysis demonstrated that the reduction of cardiovascular risk from taking a statin could be tempered by higher than anticipated statin adverse event rates, as once statin disutility was 100 times that of our basecase simulation, treat-all with high-dose statins is no longer the most cost-effective approach and at 1,000-times basecase disutility the status quo simulation which includes no risk stratification is more cost-effective than treat-all strategies. While it is possible that the true disutility from statin use is underestimated in the literature, a recent review of randomized trials of statin therapy demonstrated that only a minority of symptoms reported on statins are genuinely related to statin therapy itself [94]. While a recent analysis [95] claimed that it is unlikely that the observed increase in statin-associated side effects would lead to a disutility large enough to outweigh the benefit provided by statin therapy, in our model we found that when comparing treat-all with high-dose statins to other risk-stratification approaches, as the disutility associated with statin therapy increased, the 95% CIs for both QALYs and costs saved as compared to status quo began to overlap, indicating that as the risk of harm from statin therapy increases there is greater uncertainty regarding the cost-effectiveness of a treat-all approach to primary prevention.

### Low-Risk Population Sub-Group Analyses

We performed multiple sub-group analyses in which we modeled outcomes by different age groups, by gender, and for low-risk patients with an FRS of <5%. While treat-all with high-dose statins remains the most cost-effective approach even in lower risk populations, for low-risk women, the NNH to lead to an additional strategy-related adverse event is less than the NNT to prevent a CHD event as compared to either status quo or the ATP III strategy. While only 16% of men age 45–75 have an FRS <5%, 66% of women age 55–75 have FRS of <5%, suggesting that for a majority of women in our simulated population, more adverse events related to statin therapy would be expected to occur than CHD events prevented when utilizing a treat-all strategy. This may have significant consequences, as for example when we add a possible increased risk of intracranial bleeding with statin therapy into our model, the NNH with statins decreases further indicating that the risks of statin therapy in the low-risk population in particular may outweigh the possible benefit. The relative desirability of preventing a CHD event balanced against the detriment of incurring an adverse event (some of which are reversible) varies between cultures and depending on individual patient preferences, underscoring the difficulties inherent in providing uniform CHD prevention recommendations for all patients. In many cases, and in particular for low-risk women, it may be difficult to recommend global statin treatment, as suggested in one recent analysis contending that primary prevention strategies recommending statin therapy for low-risk populations may be misguided [96]. Rather, our findings underscore the need for, and can inform, physician-patient communication and shared decision making regarding CHD prevention. Such shared decision making should involve discussion of the available evidence, and balance a patient’s desire to avoid CHD with their viewpoint on possible statin-related side effects [97].

### 2013 ACC/AHA Lipid Guidelines vs. ATP III

The recently released ACC/AHA lipid lowering guidelines change the approach of risk-stratification from basing the initiation and escalation of statin therapy on reaching a person’s goal LDL-C level and instead focus on initiating statin therapy based on an individual’s global cardiovascular risk as calculated by the PCE. These guidelines have been met with skepticism by many in the cardiovascular community since the PCE seems to overestimate risk and if implemented worldwide could lead to over 1 billion individuals being placed on statin therapy [98,99]. Additionally a recently published analysis demonstrated that the ACC/AHA guidelines would substantially increase the proportion of individuals placed on statins as compared to the ATP III guidelines, and would increase the percentage of the age 60 to 75 year old population recommended for statin therapy from 30.4% and 21.2% under ATP III, for men and women respectively, to 87.4% and 53.6%, [35] while another recent analysis demonstrated that the use of the 2013 ACA/AHA guidelines as compared to ATP III guidelines led to more patients who were at higher risk of CHD based on CAC testing being placed on statins [100]. An additional recent analysis demonstrated that utilizing the 2013 ACC/AHA guideline recommendation of assigning statin therapy to individuals with a ASCVD risk of ≥ 7.5% was cost effective as compared to a risk cutoff of 10% and that it may even be cost-effective to prescribe statins to individuals with an ASCVD risk of as low as 3–4% which would be two-thirds of the primary prevention population [101]. Perhaps, the cost-effectiveness of the 2013 ACC/AHA guidelines is really a function of the fact that more individuals are placed on statins as compared to other risk-stratification approaches [102], and thus an extension of the findings from these recent studies may be, as we have shown in our analysis, that treating the entire primary prevention population would lead to the greatest benefit in CHD reduction. While our analysis validates the claim that the 2013 ACC/AHA guidelines would place a higher number of men and women on statins than not only the ATP III guidelines but almost any other risk-stratification strategy, it also demonstrates that in the era of generic-priced statins the ACC/AHA guidelines would be the most cost-effective risk stratification approach for women and the second most cost-effective risk stratification approach for men, second only to the JUPITER approach. However, our findings should be tempered by the fact that as statin disutility rises, the ACC/AHA guideline approach becomes less cost-effective, and at only 10-times the basecase statin disutility other strategies begin to become more cost effective.

### Health Policy Implications

Given that generic atorvastatin is now available at inexpensive wholesale prices, our results beg the consideration of potential ways to lower eligibility thresholds for statin therapy, such as policies that have recently been proposed including allowing for over-the-counter sale of statins [103], or encouraging all physicians to discuss the potential benefits and risks associated with taking statins with their age-eligible patients. Additionally, our findings further demonstrate that the focus on the initiation of statin therapy based on a person’s LDL-C level and less on their global risk may lead to many patients who might benefit from statin therapy not being treated [104]. However, the benefit of CHD reduction from an increase in statin therapy for the primary prevention of CHD needs to be balanced with exposing a greater proportion of the population to the possible harms of statin therapy. While a randomized trial evaluating all the possible strategies evaluated in our study may be a more reliable method of determining the optimal primary prevention approach to CHD that balances the benefit and harms of statin therapy in the general population, such a trial would be enormously resource-intensive. At the very least, however, our findings suggest the need for a trial to assess the benefit-harm tradeoff of statin use in low-to-medium risk patients.

The additional analysis we performed in which we compared our modeled strategies to other population based approaches aimed at the primary prevention of CHD demonstrated that large scale sustained approaches such as decreasing salt intake globally by 3 grams per day or continued smoking cessation of 1% annually for 30 years would lead to equivalent CHD reduction and QALYs saved as compared to ACC/AHA guideline implantation. While treat-all with high-dose statins would lead to a greater improvement in quality of life than salt reduction it would save less money, and thus salt reduction and smoking cessation may realize a given QALY reduction at a lower cost as compared to a treat-all approach. Placing our modeled strategies in the context of other non-statin based primary-prevention strategies not only allows for a comparison of effective primary prevention strategies, but it also highlights the importance of not placing primary prevention strategies in a vacuum. It is important to encourage multiple approaches to primary prevention, as encouraging our patients to pursue healthy lifestyles, decrease salt intake, and stop smoking, in conjunction with the judicious use of statins in appropriate patients will provide an even bigger impact in the global reduction of CHD than pursuing each of these strategies in isolation.

### Study Limitations

Our study has several limitations. Our analysis assumed that all persons aged 45–75 would be capable of undergoing CAC testing, nobody would be lost to follow up, and that there is no disutility from diagnostic tests other than increased cancer risk from radiation exposure. While statins display potent effects on lowering LDL-C and CHD risk there are many lifestyle modification strategies such as weight loss, smoking cessation, and regular exercise that can markedly decrease a person’s risk of developing CHD that were not included in our model. Our model is limited by the inclusion criteria of each of the individual risk stratification approaches we evaluated, and while it would be ideal to evaluate more than just the effect of lipid lowering on the primary prevention of CHD it is not possible to concurrently assess the effect of blood pressure reduction as only a limited number of risk stratification approaches include blood pressure as a means to estimate risk while all approaches we evaluated included LDL-C level. Additionally, our model did not specifically take into account competing risks from non-CHD diseases and thus may have underestimated health benefits and cost-savings associated with statins and aspirin. While there is likely a significant reduction in the primary prevention of ischemic stroke from statin therapy [34, 105], and while the ACC/AHA guidelines include ischemic stroke, none of the other risk stratification strategies or the FRS, which is the manner in which overall CHD risk is assessed in our model, included ischemic stroke. Additionally, to date, the use of coronary artery calcium (CAC) scores, which form the basis of the SHAPE and Texas strategies in our model, or C-reactive protein (CRP) levels, which form the basis of the JUPITER strategy in our model, have not been shown to correlate with future risk of ischemic stroke. Therefore, it is not possible to estimate ischemic stroke reduction from statin use in any other strategy evaluated in our model since we would be unable to establish a baseline risk of ischemic stroke based on a person’s initial FRS, CAC score, or CRP level. Given that the focus of our model is to understand the effects on quality of life and overall costs of utilizing various well studied and validated strategies for the primary prevention of CHD, the introduction of the primary prevention of ischemic stroke, which has not been as robustly studied and for which there are few validated risk predictive tools, would be technically very difficult and could obfuscate our overall methods and results and make our conclusions difficult to interpret. Additionally, while we only included aspirin therapy for men, and one study of the primary prevention of CHD in women demonstrated a decrease in CHD events for women over 65 years of age [106], two definitive meta-analyses demonstrated no benefit in the primary prevention of CHD for women treated with aspirin [40, 107]. We therefore did not prescribe aspirin for women in the model. Therefore, other than the effect of statins on ischemic stroke, we do not believe that background non-CHD related diseases should be different between individuals who are prescribed and not prescribed statin therapy. While some recent CAC protocols can lower the radiation dosage for CAC testing to 1 mSv, many laboratories still use protocols with higher dose, and the radiation dose of 2.3 mSv per CAC scan used in our model is similar to the dose utilized in other recent cost-effectiveness models of CAC scoring [91].

Our analysis may also underestimate costs as we did not assign costs or decreased quality of life related to patient time lost as a result of either diagnostic testing or physician visits. We utilized a PSA approach to account for the joint uncertainties of our model inputs, but were unable to include the parameters of every model input in our PSA since we only included the distribution of input parameters that were well known from the literature. Additionally, while there has been some thought that those with higher CRP levels may derive a greater benefit from statins as compared to those with lower CRP levels, we only modeled a consistent benefit of Rosuvastatin on CHD reduction as this correlation between CRP level and statin efficacy was not noted in the JUPITER trial [10]. While there is likely an interaction between FRS and CAC score, to date there has not been published data that clearly demonstrates a higher than anticipated CAC score based on age and gender for those with higher FRS. Therefore, our model does not account for an interaction between FRS and CAC score. Additionally, while there is little data regarding the benefit of statin therapy in the very-low-risk population, we modeled a decreased reduction of CHD incidence in the lower risk population from statin use by assuming a decreased reduction in CHD incidence per unit of LDL-C reduction at lower LDL-C levels. It is possible based on our sensitivity analysis that the benefit of treating all men with high-dose statins was partially a result of including aspirin use for men with low FRS, as once aspirin use was limited to those with an FRS > 10% for all interventions, JUPITER became more cost-effective than treating all with moderate-dose statins, but not high-dose statins. Our model also noted that JUPITER appeared to be more cost effective in men than in women. Perhaps since women tend to have higher CRP levels but generally lower CHD risks [27], JUPITER was not the most cost-effective risk stratification approach in women as it placed twice the number of women on statins compared to any other risk-stratification approach. Additionally, we did not model the possible effect of risk stratification on identifying very low risk individuals who might benefit from having aspirin or statin therapy discontinued. Our simulations also did not include repeat testing, as a result we may have underestimated the benefit of risk stratification-based approaches since re-testing would have likely led to an increase in the number of individuals placed on statins in these approaches.

Our model followed individuals until either their death or for 30 years after entering the model. By using a 30 year time horizon, men entering the model at age 45 years old will only be 75 years old when they are terminated out of the model. After age 75 they will still be at a high risk of accruing additional CHD events and costs. While ideally these events should be captured in our model, it is very difficult to interpret how an individual’s CHD risk today will affect their future CHD risk more than 30 years from now. The ACC/AHA guidelines for risk assessment, which include a discussion of assessing very long-term risk, state that there is insufficient evidence to date to allow us to make predictions of risk more than 30 years from now. [8] Thus, while we do believe that patients will accrue events beyond 30 years there is no evidence to guide the effect of individual risk-stratification techniques at predicting risk of CHD that far into the future. While our adherence based analysis demonstrated that even at a low adherence rate to statin and aspirin therapy of 19% for all non-CAC based strategies treat-all with high-dose statins remained the dominant strategy for both men and women, these findings should be considered in light of the limitations of our adherence-based sensitivity analysis. Modeling the concept of non-adherence is challenging and complex. Factors leading to non-adherence to medical therapy include objective adverse reactions to therapy, subjective feelings of inability to tolerate medicine, polypharmacy, and psychosocial factors related to one’s understanding and acceptance of their risk of disease as well as a necessary trust in prescribed medicines to improve one’s life in the short and long term [108–110]. As a result it is extremely difficult to capture all of the components of non-adherence in a decision analytic model. Our adherence sensitivity analysis only addresses the component of adherence related to physically taking a statin or aspirin, but not the fact that many non-adherent individuals may be less likely to see their physician regularly or undergo scheduled blood work or imaging tests. In the future, further research should be conducted to evaluate the entire spectrum of non-adherence on the cost-effectiveness of primary prevention strategies. Lastly, while we assumed our model represented the overall US population, the FRS and therefore the distribution of the PCE 10-year ASCVD risk, is based on the risk of CHD in a predominantly homogenous white male population. Therefore, the risk estimates in our model may not represent the true risk in the heterogeneous contemporary US population.

## Conclusion

In conclusion, our analysis is the first comprehensive cost-effectiveness simulation to compare multiple prevention and risk-stratification strategies for the primary prevention of CHD including treating the entire at-risk population with statins, utilizing CAC-based approaches including the SHAPE guidelines, applying CRP-based approaches such as in the JUPITER trial, following the ATP III guidelines, and implementing the ACC/AHA lipid guidelines. Under a wide range of scenarios evaluated, treating all men and women with high-dose, generic-priced statins and all men with low-dose aspirin is the most cost-effective approach for the primary prevention of CHD, while the 2013 ACC/AHA guidelines are more cost-effective than the ATP III guidelines despite placing a greater proportion of the population on statin therapy. While treating all individuals with high-dose generic statins remains the most cost-effective approach even for the lower-risk population, the reduction in CHD from statin therapy in the low-risk population, and particularly for women, must be balanced with the risk of statin-related adverse events.

## Supporting Information

S1 File Additional model inputs and results of sensitivity analyses. Table A: Distribution of 10-year risk of incident CHD per the FRS distribution versus the 10-year risk for a ASCVD event in per the Pooled Cohort Equation. Table B: Coronary heart disease and non-coronary heart disease mortality rates. Table C: FRS distribution by age and gender. Table D: baseline LDL-C level by FRS. Table E: Results of adverse event rates from a meta-analysis of high-dose statin therapy. Table F: Results from adherence based sensitivity analyses. Table G: Results from statin disutility based sensitivity analyses. Table H: Results from aspirin based sensitivity analyses. Table I: Results from CAC radiation dose based sensitivity analyses. Table J: Results When Prescribing Moderate-dose Statins in the ACC/AHA Guideline Strategy for those with a Pooled Cohort Risk of 5–7.4%.
(DOCX)
