# Supplementary material for: Strategies for Primary Prevention of Coronary Heart Disease Based on Risk Stratification by the ACC/AHA Lipid Guidelines, ATP III Guidelines, Coronary Calcium Scoring, and C-Reactive Protein, and a Global Treat-All Strategy: A Comparative--Effectiveness Modeling Study
Source: PLoS One. 2015 Sep 30;10(9):e0138092. doi: 10.1371/journal.pone.0138092 (PMC4589241; doi:10.1371/journal.pone.0138092)
Supplement: S1 File — Table A: Distribution of 10-year risk of incident CHD per the FRS distribution versus the 10-year risk for a ASCVD event in per the Pooled Cohort Equation. Table B: Coronary heart disease and non-coronary heart disease mortality rates. Table C: FRS distribution by age and gender. Table D: baseline LDL-C level by FRS. Table E: Results of adverse event rates from a meta-analysis of high-dose statin therapy. Table F: Results from adherence based sensitivity analyses. Table G: Results from statin disutility based sensitivity analyses. Table H: Results from aspirin based sensitivity analyses. Table I: Results from CAC radiation dose based sensitivity analyses. Table J: Results When Prescribing Moderate-dose Statins in the ACC/AHA Guideline Strategy for those with a Pooled Cohort Risk of 5–7.4%. (DOCX) [file pone.0138092.s001.docx]

**S1 File Table A: Distribution of 10-year risk of incident CHD per the FRS distribution versus the 10-year risk for a ASCVD event in per the Pooled Cohort Equation*^#^^**

| **Men** |  |  |  |  |
| --- | --- | --- | --- | --- |
| **FRS** | **Pooled Cohort < 5%** | **Pooled Cohort 5-7.4%** | **Pooled Cohort 7.5-9.9%** | **Pooled Cohort >10%** |
| **0-<5%** | **97.3%** | **2.5%** | **0.2%** | **0** |
| **5-7.4%** | **71.4%** | **25.5%** | **2.6%** | **0.47%** |
| **7.5-9.9%** | **21.4%** | **44.6%** | **21.5%** | **12.5%** |
| **>10%** | **7.1%** | **11.8%** | **15.4%** | **66.5%** |
| **Women** |  |  |  |  |
| **FRS** | **Pooled Cohort < 5%** | **Pooled Cohort 5-7.4%** | **Pooled Cohort 7.5-9.9%** | **Pooled Cohort >10%** |
| **0-<5%** | **85.4%** | **8.4%** | **3.0%** | **3.1%** |
| **5-7.4%** | **16.9%** | **24.3%** | **15.6%** | **43.2%** |
| **7.5-9.9%** | **5.1%** | **12.7%** | **3.8%** | **78.4%** |
| **>10%** | **21.9%** | **9.6%** | **9.1%** | **59.4%** |

*****2013 Report on the Assessment of Cardiovascular Risk: Full Work Group Report Supplement

^#^ National Cholesterol Education Program, National Heart, Lung, and Blood Institute, National Institutes of Health. (2002) Detection, Evaluation, and Treatment of High Blood Cholesterol in Adults (Adult Treatment Panel III) Final Report. NIH Publication No. 02-5215. Bethesda, MD: National Cholesterol Education Program, National Heart, Lung, and Blood Institute, National Institutes of Health.

^^^ Third Report of the National Cholesterol Education Program (NCEP) Expert Panel on Detection, Evaluation, and Treatment of High Blood Cholesterol in Adults (Adult Treatment Panel III) Final Report. (2002) Circulation 106:3143-421.

ASCVD = Atherosclerotic Cardiovascular Disease

FRS = Framingham Risk Score

**S1 File Table B**: **Coronary heart disease and non-coronary heart disease mortality rates (obtained from United States vital statistics)**

|  | **Men** |  | **Women** |  |
| --- | --- | --- | --- | --- |
| **Age** | **Background mortality** | **Mortality among CHD* patients** | **Background mortality** | **Mortality among CHD patients** |
| 45 | 0.003692 | 0.042989 | 0.002579 | 0.023478 |
| 46 | 0.004084 | 0.026081 | 0.003454 | 0.016455 |
| 47 | 0.004379 | 0.018828 | 0.002641 | 0.012674 |
| 48 | 0.004784 | 0.014798 | 0.002863 | 0.010311 |
| 49 | 0.005384 | 0.013142 | 0.003128 | 0.008694 |
| 50 | 0.005631 | 0.013159 | 0.003322 | 0.007518 |
| 51 | 0.006130 | 0.013142 | 0.003608 | 0.006625 |
| 52 | 0.006865 | 0.013159 | 0.003854 | 0.005924 |
| 53 | 0.007240 | 0.013142 | 0.004080 | 0.005358 |
| 54 | 0.007664 | 0.013159 | 0.004305 | 0.004891 |
| 55 | 0.008041 | 0.032748 | 0.004595 | 0.01352 |
| 56 | 0.008602 | 0.032791 | 0.005083 | 0.01262 |
| 57 | 0.009330 | 0.032748 | 0.005600 | 0.012034 |
| 58 | 0.009603 | 0.032791 | 0.005912 | 0.012039 |
| 59 | 0.011262 | 0.032748 | 0.006851 | 0.012034 |
| 60 | 0.011827 | 0.029558 | 0.007244 | 0.011478 |
| 61 | 0.012834 | 0.025566 | 0.008109 | 0.010815 |
| 62 | 0.014148 | 0.022522 | 0.008927 | 0.010224 |
| 63 | 0.015561 | 0.020125 | 0.009854 | 0.009694 |
| 64 | 0.016848 | 0.01819 | 0.010655 | 0.009215 |
| 65 | 0.017622 | 0.03622 | 0.011319 | 0.025387 |
| 66 | 0.019161 | 0.031253 | 0.012606 | 0.022693 |
| 67 | 0.020967 | 0.027491 | 0.013579 | 0.02052 |
| 68 | 0.022711 | 0.024542 | 0.014988 | 0.018729 |
| 69 | 0.024637 | 0.022168 | 0.016219 | 0.017229 |
| 70 | 0.026801 | 0.020777 | 0.017521 | 0.015953 |
| 71 | 0.030089 | 0.020796 | 0.020019 | 0.015266 |
| 72 | 0.032523 | 0.020777 | 0.021419 | 0.01527 |
| 73 | 0.036228 | 0.020796 | 0.023767 | 0.015266 |
| 74 | 0.038827 | 0.020777 | 0.025913 | 0.01527 |
| 75 | 0.041590 | 0.053886 | 0.027479 | 0.048867 |
| 76 | 0.046113 | 0.053817 | 0.031361 | 0.04889 |
| 77 | 0.051647 | 0.053887 | 0.034376 | 0.048867 |
| 78 | 0.056243 | 0.053816 | 0.038591 | 0.048891 |
| 79 | 0.062489 | 0.053889 | 0.042455 | 0.048867 |
| 80 | 0.067346 | 0.052009 | 0.047003 | 0.046074 |
| 81 | 0.075239 | 0.047458 | 0.051988 | 0.042238 |
| 82 | 0.082655 | 0.043616 | 0.058374 | 0.038988 |
| 83 | 0.090801 | 0.040324 | 0.065204 | 0.036199 |
| 84 | 0.099722 | 0.037474 | 0.073096 | 0.035769 |
| 85 | 0.108534 | 0.097544 | 0.079881 | 0.133714 |
| 86 | 0.121651 | 0.097627 | 0.092663 | 0.133997 |
| 87 | 0.130925 | 0.097529 | 0.100594 | 0.134285 |
| 88 | 0.146371 | 0.097424 | 0.115938 | 0.13458 |
| 89 | 0.158982 | 0.097534 | 0.127448 | 0.134882 |
| 90 | 0.168847 | 0.097421 | 0.140698 | 0.135192 |
| 91 | 0.182349 | 0.097539 | 0.154937 | 0.135509 |
| 92 | 0.195583 | 0.097532 | 0.170125 | 0.135827 |
| 93 | 0.207578 | 0.097533 | 0.185293 | 0.136166 |
| 94 | 0.218648 | 0.097421 | 0.196874 | 0.136507 |
| 95 | 0.222209 | 0.097539 | 0.215916 | 0.136856 |

CHD = coronary heart disease

**S1 File Table C: FRS distribution by age and gender***^#^

| **Men** |  |  |  |
| --- | --- | --- | --- |
| Age | Less than 10% (Standard error) | 10-20% (Standard error) | >20% (Standard error) |
| 30-39 | 91.4 % (1.5) | 7.4% (1.6) | 1.2% (.4) |
| 40-49 | 80.2% (1.7) | 16.2% (1.6) | 3.6% (.9) |
| 50-59 | 40.6% (2.4) | 52% (2.6) | 7.4 (1.3) |
| 60-69 | 8.4% (1.4) | 80.8% (1.7) | 10.8% (1.4) |
| 70-79 | 2.5% (.7) | 75.5% (2.4) | 22% (2.5) |
|  |  |  |  |
| **Women** |  |  |  |
| Age | Less than 10% (Standard error) | 10-20% (Standard error) | >20% (Standard error) |
| 30-39 | 99.9% (.1) | 0.1% (.1) | 0 (0) |
| 40-49 | 99.1% (.4) | 0.8% (.4) | 0.1% (0) |
| 50-59 | 98.2% (.6) | 1.4% (.5) | 0.4% (.2) |
| 60-69 | 91.5% (1.2) | 8.2% (1.2) | 0.3% (.2) |
| 70-79 | 55.9% (2.5) | 35.5% (2.1) | 8.6% (1.5) |

*In our simulation model, we smoothed the above distributions to create continuous cumulative probability distributions and draw samples repetitively

#Ford ES, Giles WH, Mokdad AH. (2004) The distribution of 10-year risk for coronary heart disease among US adults : findings from the National Heath and Nutrition Examination Survey III. J Am Coll Cardiol 43: 1791-6.

Abbreviations as in prior tables

**S1 File Table D: baseline LDL-C level by FRS*^#^**

| **FRS level** | **LDL<70** | **LDL 70-99** | **LDL 100-129** | **LDL 130-159** | **LDL 160-189** | **LDL > 190** |
| --- | --- | --- | --- | --- | --- | --- |
| >20% | 3.3% | 19.9% | 30.3% | 22.7% | 11.6% | 12.2% |
| 10-20% | 0.7% | 5.0% | 22.8% | 37.0% | 22.8% | 12.0% |
| 5-10% | 4.0% | 15.3% | 34.4% | 27.5% | 13.9% | 4.8% |
| < 5% | 6.6% | 24.2% | 32.9% | 22.8% | 9.7% | 3.9% |

*Keevil JG, Cullen MW, Gangnon R, McBride PE, Stein JH. (2007) Implications of cardiac risk and low-density lipoprotein cholesterol distributions in the United States for the diagnosis and treatment of dyslipidemia : data from the National Health and Nutrition Examination Survey 1999 to 2002. Circulation 115: 1363-70.

^#^In our simulation model, we assigned each simulated individual their baseline LDL levels based on the above distribution.

LDL-C = low-density cholesterol level

Other abbreviations same as in prior tables

**S1 File Table E: Results of adverse event rates from a meta-analysis of high-dose statin therapy**

| Study | Statin Evaluated | Number of Subjects | Years of Follow-up | Annual Myopathy Rate per Patient on Statin | Annual Hepatitis Rate per Patient on Statin | Annual Rhabdomyolysis Rate per Patient on Statin |
| --- | --- | --- | --- | --- | --- | --- |
| TNT* | Atorvastatin 80mg | 4,995 | 4.9 | 0.0098 | 0.0024 | 0.000082 |
| IDEAL^#^ | Atorvastatin 80mg | 4,439 | 4.8 | 0.0046 | 0.031 | 0.000094 |
| PROVE-IT^^^ | Atorvastatin 80mg | 2,099 | 2.0 | 0.0165 | 0.0165 | 0.0 |
| MIRACL^†^ | Atorvastatin 80mg | 1,538 | 0.31 | 0.0 | 0.081 | 0.0 |
| ALLIANCE** | High-dose Atorvastatin (45% on 80mg) | 954 | 4.5 | 0.0 | 0.0056 | 0.0 |
| **Meta-analysis Results** |  | **14,025** | **16.51** | **0.0074** | **0.0135** | **0.000059** |

# * LaRosa JC, Grundy SM, Waters DD, Shear C, Barter P, et al. (2005) Intensive Lipid Lowering with Atorvastatin in Patients with Stable Coronary Disease. N Eng J Med 352: 1425-35.

# #Pedersen TR, Faergeman O, Katelein JJP, Olsson AG, Tikkanen MJ, et al. (2005) High-Dose Atorvastatin vs. Usual-Dose Simvastatin for Secondary Prevention After Myocardial Infarction The IDEAL Study: A Randomized Controlled Trial. JAMA 294: 2437-45.

# ^Cannon CP, Braunwald EB, McCabe CH, Rader DJ, Rouleau JL, et al. (2004) Intensive versus Moderate Lipid Lowering with Statins after Acute Coronary Syndromes. N Eng J Med 350: 1495-504.

# †Schwartz GG, Olsson AG, Ezekowitz MD, Ganz P, Oliver MF, et al. (2001) Effects of Atorvastatin on Early Recurrent Ischemic Events in Acute Coronary Syndromes The MIRACL Study: A Randomized Controlled Trial. JAMA 285: 1711-18.

# **Koren MJ, Hunninghake DB, on behalf of the ALLIANCE Investigators. (2004) Clinical Outcomes in Managed-Care Patients With Coronary Heart Disease Treated Aggressively in Lipid-Lowering Disease Management Clinics: The ALLIANCE Study. J Am Coll Cardiol 44: 1772-9.

**S1 File Table F: Results from adherence based sensitivity analyses (95% confidence interval):**

|  | **Adherence non-CAC Strategies 52%** | | **Adherence non-CAC Strategies 36%** | | **Adherence non-CAC Strategies 19%** | |
| --- | --- | --- | --- | --- | --- | --- |
| **Men** |  |  |  |  |  |  |
|  | Total QALYs (millions) | Total Cost (billions of $) | Total QALYs (millions) | Total Cost (billions of $) | Total QALYs (millions) | Total Cost (billions of $) |
| **ATP III** | 722 (704, 738) | 479 (350, 572) | 721 (703, 738) | 482 (336, 576) | 720 (704, 738) | 489 (347, 592) |
| **Texas** | 723 (706, 740) | 490 (375, 586) | 723 (705, 741)^#^ | 487 (364, 575)^#^ | 723 (706, 740) | 487 (358, 584) |
| **SHAPE** | 723 (706, 740) | 496 (376, 590) | 723 (705, 741) | 489 (365, 585) | 723 (706, 740.5)^^^ | 486 (355, 586)^ |
| **JUPITER** | 724 (707, 740)* | 469 (341, 565)* | 723 (705, 741)* | 469 (327, 562)* | 722 (705, 739) | 474 (328, 576) |
| **ACC/AHA** | 723 (705, 740) | 459 (339, 562) | 722 (704, 740) | 468.5 (329, 564) | 721 (704, 738) | 482 (344, 581) |
| **Treat All – Moderate-dose stains** | 725 (706, 744) | 420 (205, 558) | 725 (706, 744) | 422 (198, 558) | 723.5 (706, 744) | 433 (196, 576) |
| **Treat All – High dose statins** | 727 (709, 747) | 384 (192, 523) | 726 (707, 745) | 398 (182, 538) | 724 (706, 744) | 420 (195, 561) |
| **Women** |  |  |  |  |  |  |
| **ATP III** | 390 (381, 398) | 133 (110, 157) | 390 (381, 399) | 133 (111, 158) | 391 (381, 399) | 134 (113, 158) |
| **Texas** | 390 (382, 399) | 131 (107, 154) | 390 (381.5, 399) | 131 (110, 155) | 390 (382, 400)# | 131(107.5, 154)# |
| **SHAPE** | 390 (382, 398) | 133 (112,160) | 390 (381.5, 399) | 136 (113.5, 162) | 390 (382, 399)^ | 136 (112, 161)^ |
| **JUPITER** | 390 (381, 399) | 179 (154, 203) | 390 (381, 399) | 169.5 (146.5, 194) | 389.5 (382, 399) | 160 (119, 177) |
| **ACC/AHA** | 390 (381 (399) | 127 (104, 151) | 390 (381, 399) | 129.5 (108, 154) | 390 (381, 398) | 132 (111, 156) |
| **Treat All – Moderate-dose stains** | 391 (381, 399)* | 131 (101, 161)* | 390.5 (381, 400)* | 131 (106, 157)* | 390 (381, 399) | 132 (111,156) |
| **Treat All – High dose statins** | 391 (381.5, 400) | 120 (92, 152) | 391 (382, 400) | 124 (100, 150) | 390 (381, 399) | 128 (106, 152) |

*****Cost effective compared to less expensive strategies ICER of < $50,000 per QALY gained

#Texas dominates treat-all with moderate dose statins

^SHAPE is not cost effective as compared to treat-all moderate-dose statins as the ICER is > $50,000 per QALY gained

**S1 File Table G: Results from statin disutility based sensitivity analyses (95% confidence interval):**

| **Men** | |  | |  | |  | |
| --- | --- | --- | --- | --- | --- | --- | --- |
|  | | **10-times basecase disutility** | | **100-times basecase disutility** | | **1000-times basecase disutility** | |
|  | | Total QALYs (millions) | Total Cost (billions of $) | Total QALYs (millions) | Total Cost (billions of $) | Total QALYs (millions) | Total Cost (billions of $) |
| **Status Quo** | | 717 (700, 734) | 558 (497, 619) | 713.5 (697, 731) | 558 (497, 619) | 683 (633, 718)^†^ | 558 (497, 619)^†^ |
| **ATP III** | | 722 (705, 739) | 465 (325, 565) | 716 (697, 733)^^^ | 465 (325, 565)^^^ | 651 (580, 701) | 465 (325, 565) |
| **Texas** | | 723 (706, 740) | 490 (359, 584) | 716 (698, 733) | 490 (359, 584) | 650 (572, 701) | 490 (359, 584) |
| **SHAPE** | | 723.5 (707, 740.5) | 478 (346, 579) | 715.5 (696.5, 733) | 478 (346, 579) | 636 (541, 696) | 478 (346, 579) |
| **JUPITER** | | 725 (708, 743)* | 451 (329, 540)* | 719 (701, 735.5)^#^ | 451 (329, 540)^#^ | 659 (594, 705) | 451 (329, 540) |
| **ACC/AHA** | | 723.5 (706.5, 740.5) | 431.5 (305, 536) | 708.5 (678, 732) | 431.5 (305, 536) | 559.5 (331.5, 696) | 431.5 (305, 536) |
| **Treat All – Moderate-dose stains** | | 726 (707, 745) | 399 (187, 554) | 712 (686.5, 733) | 399 (187, 554) | 575 (398, 690) | 399 (187, 554) |
| **Treat All – High dose statins** | | 729 (711, 747) | 340.5 (151, 499) | 715.5 (690.5, 737) | 340.5 (151, 499) | 580 (398, 691) | 340.5 (151, 499) |
| **Women** |  | |  |  |  |  |  |
| **Status Quo** | 390 (380, 399) | | 133 (110, 158) | 389 (380, 398)^##^ | 133 (110, 158)^##^ | 383 (371, 394)^##^ | 133 (110, 158)^##^ |
| **ATP III** | 390 (380.5, 399) | | 132 (107, 159) | 388 (379, 397) | 132 (107, 159) | 371 (351, 386) | 132 (107, 159) |
| **Texas** | 390 (381, 399) | | 130 (109, 153) | 388.5 (379, 398) | 130 (109, 153) | 371.5 (353, 387) | 130 (109, 153) |
| **SHAPE** | 390 (381, 399) | | 134 (111, 159) | 388 (378, 397) | 134 (111, 159) | 364 (339, 384) | 134 (111, 159) |
| **JUPITER** | 390.5 (381, 399) | | 193 165, 223) | 389 (379, 398) | 193 165, 223) | 372 (353, 387) | 193 (165, 223) |
| **ACC/AHA** | 390.5 (381, 399.5)** | | 122 (98, 149)** | 387 (377, 396) | 122 (98, 149) | 352 (298, 386) | 122 (98, 149) |
| **Treat All – Moderate-dose stains** | 390.5 (381, 399) | | 131 (91,173) | 383 (371, 394) | 131 (91,173) | 312.5 (217, 370.5) | 131 (91,173) |
| **Treat All – High dose statins** | 391 (382, 400) | | 113 (75, 157) | 384 (372, 395.5) | 113 (75, 157) | 314 (217, 371) | 113 (75, 157) |

*****JUPITER cost effective compared to ACC/AHA with an ICER of < $50,000 per QALY gained

**#** JUPITER cost effective compared to treat-all high-dose statins with an ICER of < $50,000 per QALY gained – JUPITER is the most cost-effective strategy at 100-times basecase statin disutility

^ATP III not cost effective compared to treat-all high dose statins as the ICER is > $50,000 per QALY gained

†Status Quo is the most cost-effective strategy at 1,000-times basecase statin disutility with an ICER compared to all other strategies of < $50,000 QALY gained

******ACC/AHA dominates treat-all moderate dose statins

##Status Quo cost effective compared to ATP III and Texas as ICER < $50,000/QALY – status quo is the most cost-effective approach at 100-times and 1,000-times basecase statin disutility

**S1 File Table H: Results from aspirin based sensitivity analyses (95% confidence interval):**

|  | **Aspirin if FRS > 10%** | | **Aspirin only if CAC > 0** | | **No Aspirin in the Model** | |
| --- | --- | --- | --- | --- | --- | --- |
|  | Total QALYs (millions) | Total Cost (billions of $) | Total QALYs (millions) | Total Cost (billions of $) | Total QALYs (millions) | Total Cost (billions of $) |
| **Status Quo** | 717 (701, 734) | 558 (497, 619)) | 717 (701, 734) | 558 (497, 619) | 717 (701, 734) | 558 (497, 619) |
| **ATP III** | 723 (705, 740) | 465 (325, 564) | 723 (705, 740) | 465 (325, 564) | 720 (703, 735) | 520 (439, 594) |
| **Texas** | 723 (707, 740) | 490 (359, 584) | 723 (706, 740) | 500 (385, 588) | 721 (703, 737) | 541 (473, 610) |
| **SHAPE** | 724 (708, 741) | 478 (346, 579) | 724 (708, 741) | 488 (369, 580) | 721 (706, 737) | 526 (444, 605) |
| **JUPITER** | 726 (708, 743)* | 451 (329, 540)* | 726 (708, 743) | 451 (329, 540) | 723 (706, 738)* | 500 (422, 577)* |
| **ACC/AHA** | 725 (707, 742)^#^ | 431 (305, 536)^#^ | 725 (707, 742) | 431 (305, 536) | 722.5 (704, 738) | 476 (377, 563) |
| **Treat All – Moderate-dose stains** | 725.5 (707,743) | 440 (285, 568) | 725.5 (707,743) | 440 (285, 568) | 722.5 (705, 739)^^^ | 490 (340, 598)^^^ |
| **Treat All – High dose statins** | 729 (711, 746) | 374 (220, 509) | 729 (711, 746) | 374 (220, 509) | 727 (708, 744) | 411 (258, 533) |

*****JUPITER cost effective compared to both ACC/AHA and treat-all moderate-dose statins with an ICER of < $50,000 per QALY gained

#ACC/AHA cost effective compared to treat-all moderate-dose statins with an ICER of < $50,000 per QALY gained **^**Treat-all moderate-dose statins not cost effective compared to ACC/AHA as the ICER is > $50,000/QALY

**S1 File Table I: Results from CAC radiation dose based sensitivity analyses (95% confidence interval):**

| **Men** |  | |  | |
| --- | --- | --- | --- | --- |
|  | **0.2 mSv/CAC Test** | | **10 mSv/CAC Test** | |
|  | Total QALYs (millions) | Total Cost (billions of $) | Total QALYs (millions) | Total Cost (billions of $) |
| **Status Quo** | 717 (701, 734) | 558 (497, 619) | 717 (701, 734) | 558 (497, 619) |
| **ATP III** | 723 (705.5, 740) | 465 (325, 565) | 723 (705.5, 740) | 465 (325, 565) |
| **Texas** | 723 (707, 740.5) | 490 (359, 584) | 722 (706.5, 740)* | 491.5 (360, 586)* |
| **SHAPE** | 724 (708, 741) | 478 (346, 579) | 711.5 (695, 728) | 487 (353, 590.5) |
| **JUPITER** | 726 (708, 743) | 451.5 (329, 540) | 726 (708, 743) | 451.5 (329, 540) |
| **ACC/AHA** | 725 (707, 742) | 431.5 (305, 536) | 725 (707, 742) | 431.5 (305, 536) |
| **Treat All – Moderate-dose stains** | 727.5 (709, 746) | 399 (187, 554) | 727.5 (709, 746) | 399 (187, 554) |
| **Treat All – High dose statins** | 730.4 (713, 748.5) | 340.5 (151, 499) | 730.4 (713, 748.5) | 340.5 (151, 499) |
| **Women** |  |  |  |  |
| **Status Quo** | 390 (382.5, 401) | 133 (110, 157.5) | 390 (382.5, 401) | 133 (110, 157.5) |
| **ATP III** | 390 (383, 401) | 132 (107, 159) | 390 (383, 401) | 132 (107, 159) |
| **Texas** | 390.5 (383, 401.5) | 130.5 (109, 153.5) | 390.5 (383, 401.5) | 131 (109, 154) |
| **SHAPE** | 390.5 (383, 401) | 134 (111, 159) | 384 (376, 394) | 137 (114.5, 162) |
| **JUPITER** | 391 (383, 393) | 193 (165, 223) | 391 (383, 393) | 193 (165, 223) |
| **ACC/AHA** | 391 (383, 402) | 122.5 (98, 149) | 390.5 (383, 402) | 122.5 (98, 149) |
| **Treat All – Moderate-dose stains** | 391 (383.5, 402) | 131 (91, 173) | 391 (383.5, 402) | 131 (91, 173) |
| **Treat All – High dose statins** | 392 (384, 402) | 113 (75, 157) | 392 (384, 402) | 113 (75, 157) |

*Texas becomes cost effective as compared to SHAPE at 10 mSv dose per CAC as the ICER is < $50,000 per QALY gained

**S1 File Table J: Results When Prescribing Moderate-dose Statins in the ACC/AHA Guideline Strategy for those with a Pooled Cohort Risk of 5-7.4% (95% confidence interval):***

|  | **Men** | | **Women** | |
| --- | --- | --- | --- | --- |
|  | Total QALYs (millions) | Total Cost (billions of $) | Total QALYs (millions) | Total Cost (billions of $) |
| **Status Quo** | 717 (701, 734) | 558 (497, 619) | 390 (382.5, 401) | 133 (110, 157.5) |
| **ATP III** | 723 (705.5, 740) | 465 (325, 565) | 390 (383, 401) | 132 (107, 159) |
| **Texas** | 723 (707, 740.5) | 490 (359, 584) | 390.5 (383, 401.5) | 130.5 (109, 153.5) |
| **SHAPE** | 724 (708, 741) | 478 (346, 579) | 390.5 (383, 401) | 134 (111, 159) |
| **JUPITER** | 726 (708, 743) | 451.5 (329, 540) | 391 (383, 393) | 193 (165, 223) |
| **ACC/AHA** | 725 (707, 742) | 426 (302.5, 532) | 391 (382, 400) | 121 (96, 149.5) |
| **Treat All – Moderate-dose stains** | 727.5 (709, 746) | 399 (187, 554) | 391 (383.5, 402) | 131 (91, 173) |
| **Treat All – High dose statins** | 730.4 (713, 748.5) | 340.5 (151, 499) | 392 (384, 402) | 113 (75, 157) |

*Cost-effectiveness of ACC/AHA as compared to other strategies unchanged as compared to basecase simulation for both men and women

ACC/AHA = 2013 American College of Cardiology and American Heart Association Guidelines on Lipid Lowering Therapy

CAC = coronary artery calcium score

CI = confidence interval

QALY = Quality Adjusted Life Years

SHAPE = Screening for Heart Attack Prevention and Education

ATP III = Adult Treatment Panel III

JUPITER = Justification for the Use of Statins in Primary Prevention

mSv = milisieverts
